# Integrative analysis of genetic variability and functional traits in lung adenocarcinoma epithelial cells via single-cell RNA sequencing, GWAS, bayesian deconvolution, and machine learning

**DOI:** 10.1007/s13258-025-01621-2

**Published:** 2025-02-24

**Authors:** Chenggen Gao, Jintao Wu, Fangyan Zhong, Xianxin Yang, Hanwen Liu, Junming Lai, Jing Cai, Weimin Mao, Huijuan Xu

**Affiliations:** 1 Jiangxi medical college, Nanchang university, Nanchang, China; 2 NHC Key Laboratory of Personalized Diagnosis and Treatment of Nasopharyngeal Carcinoma, Jiangxi Cancer Hospital, The Second Affiliated Hospital of Nanchang Medical College, Nanchang, China; 3 Department of Thoracic Surgery, The Third Affiliated Hospital of Zhejiang Chinese Medicine University, Hangzhou, China; 4 Department of Clinical Laboratory, Hangzhou TCM Hospital Affiliated to Zhejiang Chinese Medical University, Hangzhou, China; 5 The fifth affiliated hospital of jinan university, Heyuan, Guangdong China; 6 Department of general surgery, The Third Affiliated Hospital of Zhejiang Chinese Medical University, Nanchang, China; 7 Ganjiang New District Hospital of the First Affiliated Hospital of Nanchang University, Nanchang, China; 8 Department of Thoracic Surgery, Jiangxi Cancer HospitalJiangxi Province, Nanchang, China; 9 Lung cancer center, The second affiliated hospital of Nanchang University, Nanchang, China

**Keywords:** Lung adenocarcinoma, Single-cell RNA sequencing, GWAS, Bayesian deconvolution, Machine learning

## Abstract

**Background:**

Lung adenocarcinoma remains a leading cause of cancer-related mortality worldwide, characterized by high genetic and cellular heterogeneity, especially within the tumor microenvironment.

**Objective:**

This study integrates single-cell RNA sequencing (scRNA-seq) with genome-wide association studies (GWAS) using Bayesian deconvolution and machine learning techniques to unravel the genetic and functional complexity of lung adenocarcinoma epithelial cells.

**Methods:**

We performed scRNA-seq and GWAS analysis to identify critical cell populations affected by genetic variations. Bayesian deconvolution and machine learning techniques were applied to investigate tumor progression, prognosis, and immune-epithelial cell interactions, particularly focusing on immune checkpoint markers such as PD-L1 and CTLA-4.

**Results:**

Our analysis highlights the importance of genes like SLC2A1, which regulates glucose metabolism and correlates with tumor invasiveness and poor prognosis. Immune-epithelial interactions suggest a suppressive tumor microenvironment, potentially hindering immune responses. Additionally, machine learning models identify core prognostic genes such as F12, GOLM1, and S100P, which are significantly associated with patient survival.

**Conclusions:**

This comprehensive approach provides novel insights into lung adenocarcinoma biology, emphasizing the role of genetic and immune factors in tumor progression. The findings support the development of personalized therapeutic strategies targeting both tumor cells and the immune microenvironment.

**Supplementary Information:**

The online version contains supplementary material available at 10.1007/s13258-025-01621-2.

## Introduction

Lung cancer remains one of the leading causes of cancer-related deaths worldwide, with lung adenocarcinoma, in particular, exhibiting a persistently high incidence among non-smoking-related lung cancers. Each year, over 1.8 million people die from lung cancer, and lung adenocarcinoma is especially difficult to treat due to its complex biological behavior and the individual variability in treatment response (Wu et al. 2021; Shi et al. 2023). A major challenge in the clinical treatment of lung adenocarcinoma is its marked heterogeneity, which manifests not only within tumor cells but also across various levels of the tumor microenvironment (McKay et al. 2017; Hirz et al. 2023; Mao et al. 2024).

Single-cell RNA sequencing (scRNA-seq) technology provides a unique perspective, allowing us to precisely uncover this heterogeneity at the single-cell level (Zhang et al. 2023a, b; Dai et al. 2019). By analyzing gene expression data from individual cells, researchers can identify the presence of different cell populations within the tumor, including tumor cells, immune cells, fibroblasts, and endothelial cells (Kim et al. 2020; Ren et al. 2024; Feng 2023). This intricate network of cells plays a critical role in the progression, metastasis, and therapeutic response of lung adenocarcinoma (Zhang et al. 2023a, b; Passaro et al. 2022; Sinjab et al. 2021). Particularly, single-cell technologies can reveal dynamic changes in the tumor microenvironment, such as how immune cells are recruited and manipulated to aid tumor evasion, and how they either fight against or support tumor growth (Cords et al. 2024; Qiao et al. 2024).

Furthermore, genome-wide association studies (GWAS) have identified numerous genetic loci associated with lung adenocarcinoma risk (Marjanovic et al. 2020; Montégut et al. 2024). However, the functional roles of these loci—how they influence gene expression and cellular behavior—cannot be elucidated by GWAS alone (Xiang et al. 2024; Zhang et al. 2024). By integrating scRNA-seq data with GWAS results, we can gain deeper insights into how these genetic variations affect gene expression in different cell types, thereby revealing their biological significance (Li et al. 2022; Zhang et al. 2023a, b). For example, specific genetic markers may regulate signaling pathways in immune cells, influencing their behavior in the tumor microenvironment—an understanding crucial for developing novel immunotherapeutic strategies (Palermo et al. 2023; Dhainaut et al. 2022).

In this study, we employed Bayesian deconvolution, an advanced statistical method that allows us to accurately dissect gene expression data from individual cell types within mixed tissue samples. By combining scRNA-seq data from lung adenocarcinoma patients with extensive GWAS data, this technique enables us to directly observe the expression patterns and impacts of specific genetic variants in different cell types. This precision is particularly valuable for unraveling the molecular mechanisms underlying complex diseases like lung adenocarcinoma. Through Bayesian deconvolution analysis, we identified cell populations affected by specific genetic variants, and explored how these variants contribute to lung adenocarcinoma progression and prognosis. Special attention was given to key genes and pathways involved in intercellular signaling, cell cycle regulation, and cell death.

## Methods

### Single-cell RNA sequencing data analysis

To explore cellular heterogeneity and its role in cancer progression, we performed a comprehensive analysis of single-cell RNA sequencing (scRNA-seq) data using the Seurat package (Butler et al. 2018). First, we loaded the scRNA-seq data object af and conducted a thorough quality control (QC) assessment. The QC metrics included the number of detected features (nFeature_RNA), the total RNA counts (nCount_RNA), the percentage of mitochondrial genes (percent.mt), and the percentage of ribosomal genes (percent.rb). These metrics were visualized through violin plots, and scatter plots were used to show correlations between the QC metrics to ensure data reliability.Subsequently, the data were normalized using the LogNormalize method, and 2,000 highly variable genes were identified. These genes, which are highly expressed in some cells and lowly expressed in others, are critical for understanding functional differences between cell types. Principal component analysis (PCA) and t-distributed stochastic neighbor embedding (tSNE) were then applied to reduce data complexity and visualize the distribution of different cell types. In addition, batch effects were corrected using the Harmony method, minimizing the impact of technical and biological variability on the analysis results.Next, we performed cell clustering at various resolutions using the FindClusters function, optimizing the resolution parameters to achieve the best clustering outcomes. Cell annotation was conducted with the SingleR package and human primary cell reference data from the celldex dataset, identifying the identity of each cell cluster. Manual annotation was further applied to confirm cell types, using known marker gene lists for cell identification, which were visualized through heatmaps and dot plots.For the subsequent BayesPrism deconvolution analysis, a random subset of cells from each cell subpopulation was selected to generate the single-cell input file. Genes with high expression but low cell-type specificity, such as ribosomal protein genes and mitochondrial genes, were excluded, along with sex chromosome genes and lowly expressed genes. Finally, the pre-processed single-cell matrix and cell annotation information were saved, providing a foundation for further analysis. We first performed quality control and normalization of the single-cell RNA sequencing data. Using the Seurat package, we preprocessed the raw data by filtering out low-quality cells and identifying highly variable genes. Dimensionality reduction was carried out using PCA and t-SNE, allowing us to map the data into a low-dimensional space and identify distinct cell populations, which were then annotated to ensure the accuracy of each cell group. Based on this foundation, we extracted the gene expression profiles of each cell population, providing critical inputs for subsequent Bayesian deconvolution and GWAS data integration.

### Integration of single-cell RNA sequencing and GWAS Data

Next, we processed the GWAS data by filtering for genetic variants associated with lung adenocarcinoma and extracting key statistical information, including locus details, effect sizes, and standard errors. To ensure compatibility with the scRNA-seq data, we preprocessed the GWAS data using the VariantAnnotation package, preparing it for the subsequent scPagwas analysis.This study aims to integrate single-cell RNA sequencing (scRNA-seq) data with genome-wide association study (GWAS) (Cui et al. 2023) data to explore the relationship between gene expression and phenotypes in lung cancer. The analysis workflow consists of data loading and preprocessing, GWAS data handling, integration analysis, and result visualization.First, we loaded and preprocessed the previously normalized, dimensionally reduced, and clustered scRNA-seq data. The data were processed using the Seurat package to ensure accurate annotation and gene expression information for each cell type. Next, we processed the GWAS data using the VariantAnnotation package to read GWAS data files and convert them into a format suitable for scPagwas analysis. Key fields such as chromosomal location, variant ID, standard error (SE), effect size (ES), and other essential fields were extracted and saved as summary statistics. After necessary format conversions, including information on minor allele frequency (MAF), the data were prepared for use in scPagwas analysis.We then performed the integrated analysis using the scPagwas package (Ma et al. 2023), which combines scRNA-seq and GWAS data. The main analysis function, scPagwas_main, was executed by specifying the file paths for the GWAS data and single-cell data, setting the analysis parameters, and enabling multithreading for efficiency. This step allowed the identification of genetic variants associated with specific cell types, exploring their roles across different cell types and their impact on phenotypes.Finally, the results of the integrated analysis were visualized using the built-in functions of the scPagwas package. Visualizations included bar plots based on the Bootstrap method, distribution plots of cell types by TRS scores, and scatter plots of trait-associated genes. These graphical representations help intuitively understand how genetic variants influence the function and phenotype of specific cell types, providing crucial insights for further research.

### TCGA data download, processing, and differential gene expression analysis

In this study, we utilized the TCGAbiolinks package (Colaprico et al. 2016) to download and process data from The Cancer Genome Atlas (TCGA) for the lung adenocarcinoma (TCGA-LUAD) project. The specific steps included retrieving gene expression quantification data using the GDCquery function, followed by downloading and preparing the data with the GDCdownload and GDCprepare functions. Gene expression matrices, gene annotation information, and clinical data were extracted and saved as CSV files, while simple nucleotide variation (SNV) data were downloaded and saved as Rdata files. These steps provided complete and accurate gene expression, annotation, and clinical data, forming the foundation for subsequent bioinformatics analyses.Next, differential expression analysis was conducted using the DESeq2 package (Love et al. 2014) on the gene expression data downloaded from the TCGA database. The preprocessing steps involved reading and organizing the gene expression matrix and clinical information, deduplicating the data, and extracting normal and tumor samples to generate sample grouping information. A DESeqDataSet object was constructed and normalized, after which differential expression analysis was performed using the DESeq function. This analysis calculated the log2FoldChange and adjusted p-values (padj) for each gene, identifying significantly differentially expressed genes.To visualize the results, we used the ggplot2 package to create a volcano plot of the differentially expressed genes. In this plot, the x-axis represents log2FoldChange, and the y-axis represents -log10(padj), with significantly upregulated and downregulated genes highlighted in distinct colors. Important genes were further filtered and annotated in the plot.Additionally, heatmaps were generated using the SummarizedExperiment and sechm packages to display the expression patterns of significantly upregulated and downregulated genes across different samples. The gene expression data were log2-transformed and standardized across both genes and samples to better illustrate differential expression patterns.

### BayesPrism deconvolution analysis

The Bayesian deconvolution method was a key step in our analysis. In this phase, we integrated scRNA-seq and GWAS data using deconvolution tools such as BayesPrism. Specifically, we extracted gene expression data from each cell population and applied Bayesian deconvolution techniques to accurately estimate the gene expression characteristics of different cell types within mixed tumor samples. This approach allowed us to gain detailed insights into gene expression changes across various cell populations and their associations with lung adenocarcinoma progression.In the Bayesian deconvolution process, the scPagwas package played a crucial role. This tool integrates GWAS data with scRNA-seq data and calculates Trait Risk Scores (TRS) to identify genetic variants associated with specific cell types. For instance, scPagwas analysis enabled us to detect significant expression of certain genetic variants in distinct cell populations and explore their potential links to lung adenocarcinoma.In this study, BayesPrism (Chu et al. 2022) was used to perform deconvolution analysis on single-cell RNA sequencing (scRNA-seq) data and bulk transcriptomic data. The analysis began by reading the preprocessed single-cell count matrix, cell annotation file, and bulk transcriptomic count matrix data. Next, the deconvolution parameters were set, including the bulk expression matrix, single-cell matrix, cell annotation information, and setting the key parameter to NULL in the absence of malignant cells. Additionally, the number of cores used for the analysis was set to 10 (ncores = 10).For each bulk sample, a gene expression consistency plot between the bulk and single-cell data was generated, with protein-coding genes selected for the deconvolution analysis. A Prism object was then created, containing both the reference single-cell data and the mixed bulk data. Parameters such as outlier clipping (outlier.cut = 0.01) and outlier fraction (outlier.fraction = 0.1) were adjusted to manage the influence of outliers on the analysis.The deconvolution was then performed using BayesPrism, extracting the posterior mean proportions of different cell types present in the bulk samples. These steps allowed us to dissect the proportions of various cell types in the bulk samples, providing deeper insights into the cellular composition and complexity of the tumor microenvironment.

### Cox regression and survival analysis

In this study, Cox regression and survival analysis were employed to assess the impact of gene expression on the prognosis of lung adenocarcinoma (TCGA-LUAD) patients. First, the gene expression matrix and clinical data were read from CSV files and preprocessed by removing normal samples and integrating survival time and status information. Cox regression analysis was then performed for each gene, calculating the hazard ratio (HR), 95% confidence interval (CI), and p-value.Patients were stratified into high-risk and low-risk groups based on the median gene expression levels, and survival differences between these groups were evaluated using Kaplan-Meier survival curves and log-rank tests. Finally, survival curves and risk tables were plotted to illustrate the influence of different gene expression levels on patient prognosis, providing important insights for further research into the prognostic markers of lung adenocarcinoma.

### Infiltration differences of convolutional cells between cancer and adjacent normal tissues

Using the rstatix and ggplot2 packages, we analyzed and visualized the significant differences in the proportions of different cell types between groups. First, the gene expression matrix and sample grouping information were loaded, ensuring consistency in sample names between the two data files, and then integrated with the sample grouping information. Next, the data were reshaped into a long format using the reshape2 package, containing sample IDs, cell types, and cell type proportions (Theta).We then applied t-tests for each cell type between groups using the rstatix package, and p-values were adjusted using the Bonferroni method to identify significant differences. Finally, we visualized the results using violin plots and box plots created with the ggplot2 package, adding significance markers to display the differences between groups. Significant results and markers for each cell type were added to the plots, which were saved as PDF files.These steps allowed us to visually present and understand the significant differences in cell type proportions between groups, providing a solid basis for further research.

### Differential analysis of convolutional cell groupings

First, the gene expression matrix and sample grouping information were read from CSV files, and normal samples were excluded. Next, the proportions of the specified cell types were extracted from the post-convolution cell proportion data. Samples were then divided into high-risk and low-risk groups based on the median or a specified threshold of cell type proportions.A DESeqDataSet object was created, and the data were normalized. Differential expression analysis was performed using the DESeq function, calculating the log2FoldChange and adjusted p-values (padj) for each gene. A volcano plot was generated to visualize the differentially expressed genes.Following this, the tidyestimate package was used to calculate the immune score, stromal score, estimate score, and purity of the samples. These scores were combined with sample grouping and cell proportion information. The Wilcoxon test was applied to assess significant differences between groups, and violin plots and box plots with significance markers were drawn to illustrate these differences.Finally, the ComplexHeatmap package was employed to create a heatmap, displaying the expression patterns of significantly different genes between groups.

### Clinical trait analysis of specified convolutional cell groupings

First, sample grouping information, clinical data, and convolutional cell proportion data were collected, ensuring consistent sample names across all data files, and selecting the proportion data for the specified cell type. Next, based on a predefined list of comparison groups (including clinical features such as Stage, Age, T, N, and M), samples were grouped according to each clinical trait.For each clinical feature, a DataFrame was created containing cell proportions and clinical trait information, with the levels of each clinical trait ordered according to a predefined sequence. Box plots were then generated using the ggboxplot function to visualize the cell type proportions across different clinical trait groups. Statistical significance tests were performed using the stat_compare_means function, and significance markers were added to highlight differences between groups.

### Immune therapy analysis of specified convolutional cells

In this study, the reticulate package was used to call the Python-based Tumor Immune Dysfunction and Exclusion (TIDE) tool within the R environment, analyzing and visualizing the functional states of different immune cell types within the tumor microenvironment. First, the Python environment was set up and activated using the reticulate package, followed by loading the necessary Python libraries and data.Next, the gene expression data were imported and analyzed using the TIDE tool to generate scores related to immune evasion. These TIDE analysis results were then integrated with the immune grouping information of the samples. To visualize the functional state differences of various immune cell types between the high and low immune groups, violin plots with significance markers were generated using the ggpubr package.

### Drug response prediction for specified convolutional cells

In this study, the oncoPredict package (Maeser et al. 2021) was used to predict and visualize drug responses for immune cell types within the tumor microenvironment. First, the gene expression matrix and sample grouping information were obtained, ensuring consistency in sample names between the two datasets. Next, gene expression data and drug response data from the GDSC2 database were loaded and normalized. Using the oncoPredict package, drug response phenotypes were calculated for each sample.The prediction results were then integrated with the immune grouping information of the samples, followed by differential significance analysis. The Wilcoxon test was applied to assess significant differences between groups, and a heatmap was generated using the pheatmap package to visualize drug responses for significantly different drugs across groups.Additionally, box plots were generated using the ggpubr package to illustrate the drug response differences between immune groups for each drug, with significance differences clearly marked.

### SNV analysis of specified convolutional cells

In this study, the maftools package was used to analyze and visualize the mutation characteristics of different immune cell types in tumor samples. First, sample grouping information was read from CSV files, and sample IDs were processed to ensure uniqueness and consistency. Next, the MAF (Mutation Annotation Format) file was loaded and integrated with the sample grouping information, generating a MAF object that included clinical data. A color scheme was defined to distinguish between the immune cell groupings.The oncoplot function was used to visualize the top 30 mutated genes, showing mutation patterns across different groups. The plotmafSummary function was then used to generate a summary report of the MAF file, providing an overview of global mutation characteristics.Next, mutation differential analysis was conducted. Samples were divided into two groups (high-expression and low-expression) based on the specified immune cell type, and the mafCompare function was employed to compare the differential mutated genes between the two groups. The results of the differential mutation analysis were saved as a CSV file.Finally, the significantly different mutated genes were visualized using the coBarplot and forestPlot functions, generating bar plots and forest plots to provide a clear and intuitive representation of mutation differences between the groups.

### WGCNA analysis of convolutional cells

In this study, the WGCNA package was used to perform a weighted gene co-expression network analysis on gene expression data to identify gene modules associated with lung adenocarcinoma (LUAD). First, the gene expression matrix, immune infiltration results, and sample type information were loaded and preprocessed, including the removal of duplicate samples and low-expression genes. The top 10,000 genes were selected for analysis.Next, sample clustering was performed on the gene expression data to detect and remove outlier samples. The immune infiltration results and clinical trait data were then integrated. A soft-threshold power was selected to compute the scale-free network topology, and the optimal soft-threshold power was determined. The adjacency matrix between genes was then calculated and transformed into a topological overlap matrix (TOM). Hierarchical clustering was applied to identify gene modules.For each gene module, module eigengenes were calculated and clustered to merge similar modules, generating the final module partition. The correlation between each module eigengene and clinical traits was then calculated, and a module-trait correlation heatmap was created to visualize the strength of the association between each module and LUAD.Finally, the module membership (MM) of each gene within its module and the gene significance (GS) were calculated. Scatter plots were generated to visualize the relationship between MM and GS. Through these steps, gene modules significantly associated with LUAD were identified, providing important insights and potential therapeutic targets for further research.

### Venn diagram construction and core gene selection

In this study, we integrated multiple gene expression datasets and performed differential expression analysis to identify genes with significant changes under different conditions. A Venn diagram was then constructed to compare and visualize the overlap between these gene sets, allowing us to identify common differentially expressed genes across conditions.Subsequently, these intersecting genes were further analyzed to screen for core genes. Functional enrichment analysis and network analysis were employed to determine which genes play a critical role in the immune response of lung adenocarcinoma epithelial cells. These core genes may serve as potential therapeutic targets, providing important insights for further investigation into their roles in cancer progression.

### Association between core genes and convolutional cells

First, the convolutional cell matrix and gene expression matrix were loaded, and the gene expression matrix was log-transformed. Next, the sample information file was read, and normal control samples were excluded. The convolutional cell matrix and gene expression matrix were then integrated by matching samples.To improve computational efficiency, multicore parallel computing was employed. A cluster was created, and a processing function was established for each pair of genes and cell types. In this function, the Spearman correlation between each gene and cell type was calculated along with the corresponding p-values. Multiple testing correction was applied to the p-values, and if the corrected p-values were significant, a scatter plot of the correlation was generated.

### Screening hub genes using 101 machine learning algorithm combinations

In this study, the Mime1 package (Liu et al. 2024) and other related R packages were utilized for various analyses. The Mime1 package integrates 101 combination algorithms based on five machine learning methods: Lasso, randomForestSRC, Enet (Elastic Net), CoxBoost, and SuperPC. These combinations were used to construct and evaluate multiple predictive models.First, the ML.Dev.Prog.Sig function was applied to build prognostic prediction models based on a candidate gene list and training data. The models were validated using different datasets, generating C-index distribution plots and survival curves for the prognostic prediction models. Next, the ML.Corefeature.Prog.Screen function was used to screen for core feature genes, and various methods were employed to generate Upset plots and rank plots of the selected genes, analyzing their correlation and survival curves.This study systematically constructed and evaluated multiple predictive models, analyzed immune infiltration patterns, and identified core feature genes. The findings provide valuable insights and data support for further biomarker discovery and clinical applications.

### Cell culture

The lung adenocarcinoma cell lines PC9 and H1650 were purchased from the Chinese Academy of Sciences Cell Bank (Shanghai, China). PC9 and H1650 cells were cultured in RPMI-1640 medium (Gibco, NY) supplemented with 1% penicillin-streptomycin (Beyotime, Nanjing, China) and 10% fetal bovine serum (Gibco, NY). The cells were incubated in a 5% CO₂ atmosphere at 37 °C.

### Cell transfection

To transiently knock down the expression of SLC2A1, siRNA sequences targeting SLC2A1 were designed (S1: 5′- GCCUGUGUAUGCCACCAUUTT-3′ (sense) and 5′- AAUGGUGGCAUACACAGGCTT-3′ (anti-sense); S2: 5′- GCAUCAACGCUGUCUUCUATT-3′ (sense) and 5′- UAGAAGACAGCGUUGAUGCTT-3′ (anti-sense); S3: 5′- GGAUGUCCUAUCUGAGCAUTT-3′ (sense) and 5′- AUGCUCAGAUAGGACAUCCTT-3′ (anti-sense)), and purchased from GenePharma (Shanghai, China). Transfections were performed using the siRNA-mate plus transfection reagent (GenePharma, China) according to the manufacturer’s instructions.

### Western blotting

Western blotting was used to detect the expression of SLC2A1, P53, P21, and β-catenin. The primary antibodies used in this study included anti-SLC2A1 (Proteintech, 66290-1-Ig), anti-P53 (Proteintech, 10442-1-AP), anti-P21 (Proteintech, 10355-1-AP), and anti-β-catenin (Proteintech, 66009-1-Ig). Primary antibodies were diluted with dilution buffer (Solarbio, China) and incubated overnight at 4 °C. The next day, after incubation with secondary antibodies, protein bands were detected using an imaging system (Bio-Rad, USA), and protein intensities were quantified using ImageJ.

### Invasion and migration assays

Transfected cells were resuspended in serum-free RPMI-1640 medium and seeded at a density of 5 × 10⁴ cells per well in the upper chamber of a Transwell system (NEST, China). The lower chamber contained 700 µl of medium supplemented with 10% FBS as a chemoattractant. After incubation for 24–48 h, cells in the upper chamber were removed with a cotton swab. The invading cells in the lower chamber were fixed with 4% paraformaldehyde and stained with 0.5% crystal violet for 30 min. Images were captured under an inverted microscope (Leica, Germany) and quantified.

### Cell counting Kit-8 (CCK-8) assay

Transfected cells were resuspended and seeded at a density of 2000 cells per well in a 96-well plate with 100 µl of complete medium. Four replicates were set for each cell type. Absorbance was measured at 450 nm at 24, 48, 72, 96, and 120 h.

### Colony formation assay

Transfected cells were resuspended and seeded at a density of 2000 cells per well in a 6-well plate, with three replicates for each condition, and cultured for 2 weeks. After washing with PBS, cells were fixed with 4% paraformaldehyde for 30 min and stained with 0.1% crystal violet for 30 min. Colonies were washed, air-dried, and analyzed using ImageJ for colony formation and relative colony numbers.

### Data analysis

Statistical analysis and graphical representation were performed using GraphPad Prism 10 software. Independent t-tests or one-way ANOVA were used to compare the means between two groups or multiple groups, respectively. All data analyses were performed based on R 4.1.3, with *p* < 0.05 considered statistically significant.

## Results

### Single-cell data processing

**Fig. 1:**
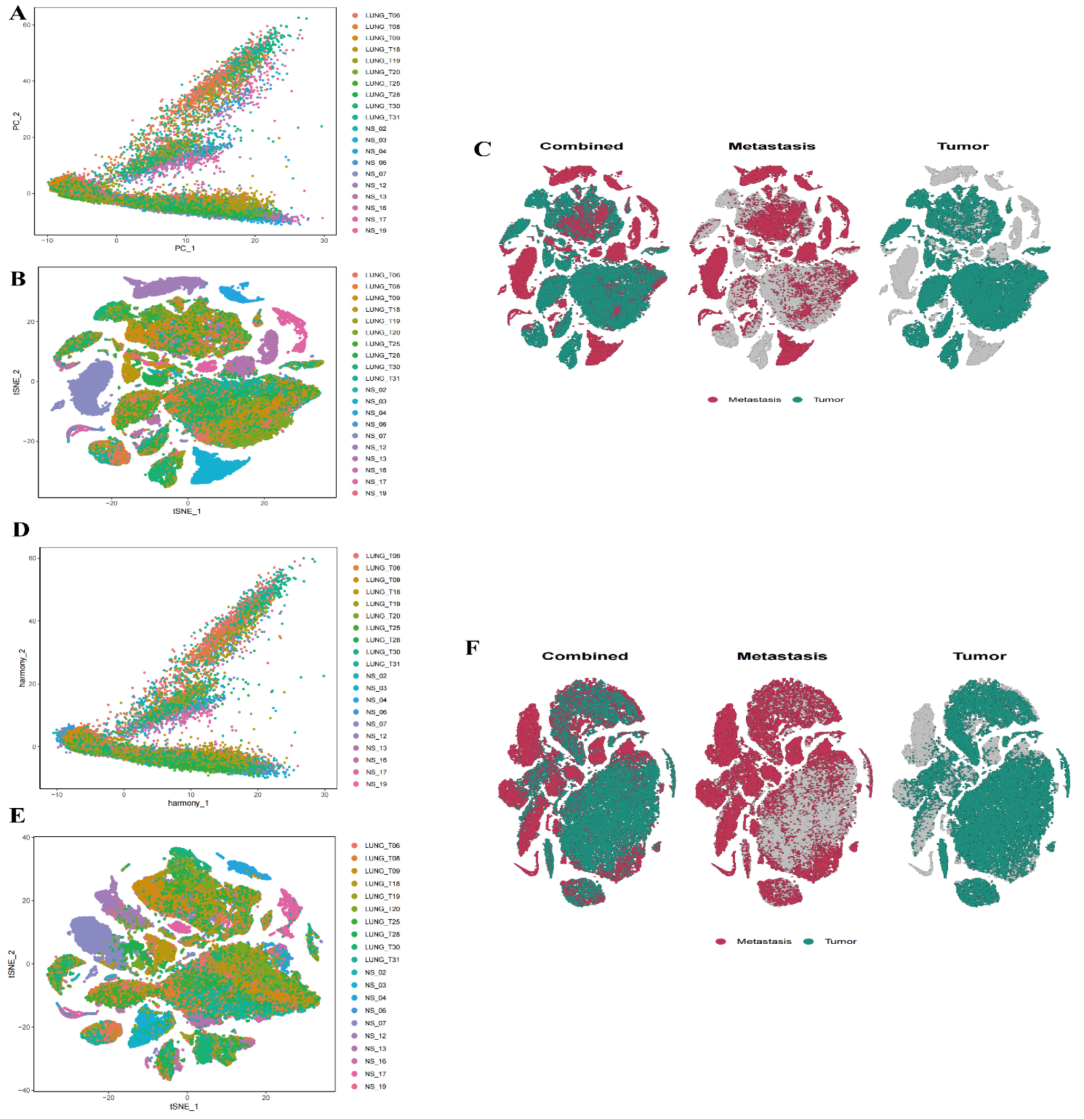
Dimensionality Reduction and Batch Effect Correction **A** PCA plot showing the distribution of the first two principal components (PC_1 and PC_2). Cells from different samples are distributed across the space, with colors representing different samples. **B** t-SNE plot illustrating the clustering of high-dimensional data in low-dimensional space without batch effect correction. Cells from different samples form distinct clusters in the t-SNE plot. **C** t-SNE plot showing the distribution of tumor and metastatic cells. Tumor cells and metastatic cells are labeled in green and red, respectively, demonstrating their distribution in low-dimensional space in both combined and separated views. **D** PCA plot after Harmony batch effect correction, where the distribution of data points is tighter and more consistent, indicating successful batch effect correction. **E** t-SNE plot after Harmony batch effect correction, showing more concentrated clustering of data points, further demonstrating the effective elimination of batch effects. **F** t-SNE plot of tumor and metastatic cell distributions after Harmony correction, showing their distribution under different conditions in both combined and separated views, indicating the improved separation after batch effect correction

We conducted a quality control (QC) analysis to assess the number of RNA features, RNA counts, mitochondrial gene proportion, and red blood cell gene proportion for each sample (Supplementary Fig. 1A). The results showed a correlation of 0.24 between mitochondrial gene proportion and RNA count, -0.01 between red blood cell gene proportion and RNA count, and 0.91 between RNA feature number and RNA count (Supplementary Fig. 1B). According to QC standards, the mitochondrial gene proportion should typically be between 5% and 20%, the red blood cell gene proportion should be minimized, the number of detected genes per cell should range from 200 to 2,500, and RNA counts should range from 500 to 50,000.We identified 2,000 highly variable genes, which are highly expressed in some cells and lowly expressed in others. The top 10 highly variable genes are labeled in the figure (Supplementary Fig. 1C). These highly variable genes were used for subsequent dimensionality reduction analysis. Dimensionality reduction was performed using PCA and t-SNE methods. The PCA plot showed the distribution of the first two principal components, revealing distinct distribution patterns across samples (Fig. 1A). The t-SNE plot further illustrated the clustering of high-dimensional data in low-dimensional space (Fig. 1B), with clear clustering of cells from different samples (Fig. 1C).To eliminate batch effects, the Harmony algorithm was applied for batch effect correction. The corrected PCA and t-SNE plots (Fig. 1D, E) demonstrated effective elimination of batch effects, with tighter and more consistent cell distributions in low-dimensional space. The separated and combined t-SNE plots showed the distribution of tumor and metastatic cells under different conditions (Fig. 1F), further validating the effectiveness of the batch effect correction.We analyzed the feature genes of the top 20 principal components (PCs) using PCA, visualizing the gene expression patterns of these components in a heatmap (Supplementary Fig. 2A). The JackStraw test results indicated significant differences among the top 20 principal components (Supplementary Fig. 2B). The elbow plot helped determine the optimal number of principal components for clustering analysis, leading to the selection of the top 7 principal components for further analysis (Supplementary Fig. 2C).Based on the top 7 principal components, a K-nearest neighbor (KNN) graph was constructed using Euclidean distance, and cell clustering was performed with different resolutions. The clustering results were visualized using a Clustree plot (Supplementary Fig. 2D), clearly displaying the hierarchical relationships between clusters at different resolutions.

### Single-cell RNA sequencing analysis and cell type annotation

**Fig. 2:**
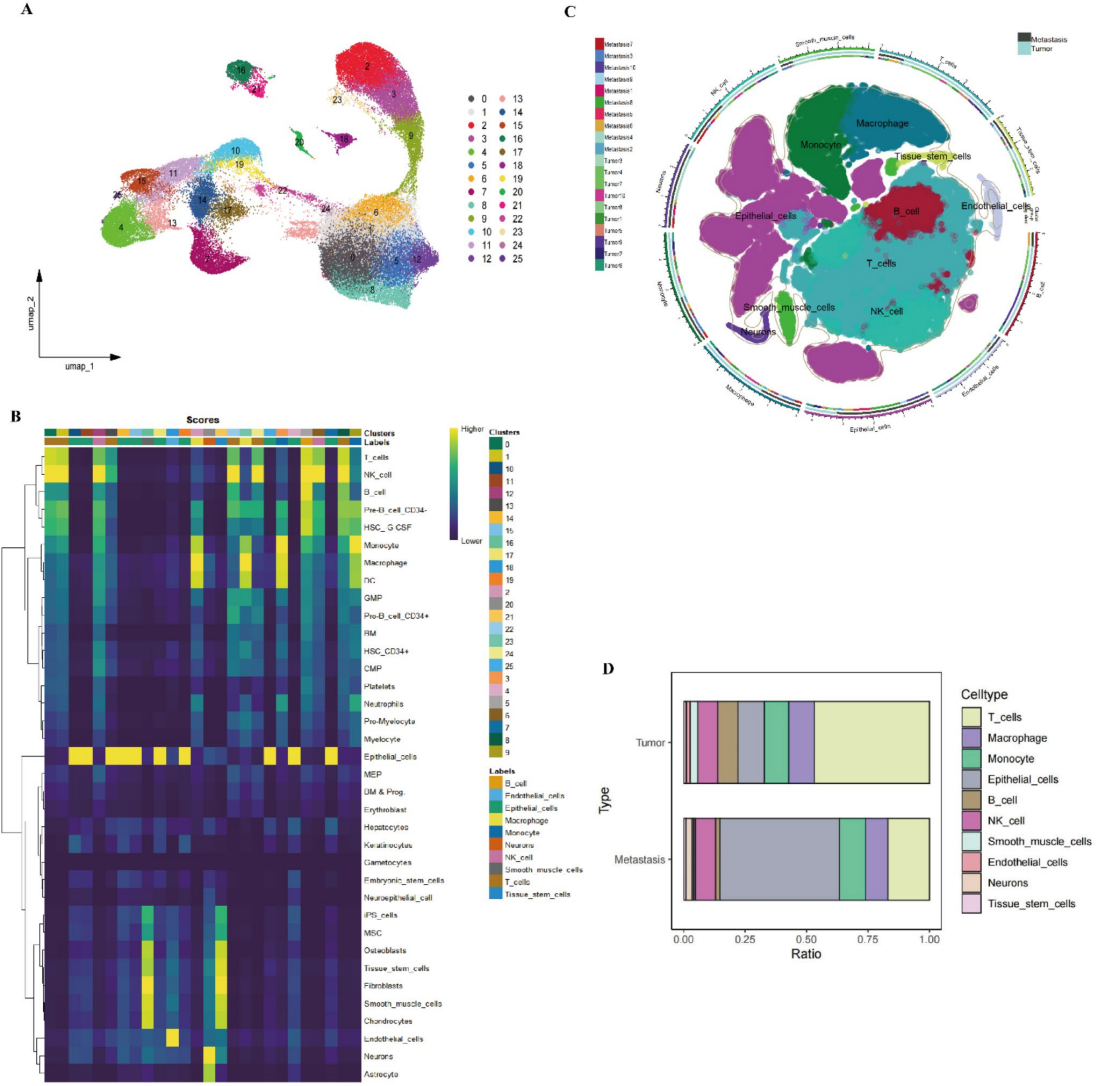
UMAP Dimensionality Reduction, Cell Type Annotation, and Proportion Analysis of Single-Cell RNA Sequencing Data. **A** UMAP dimensionality reduction of single-cell RNA sequencing data, dividing all cells into 25 distinct clusters. Each color represents a different cell cluster, with each cluster corresponding to a specific cell population. The UMAP plot shows the distribution of these clusters in low-dimensional space, illustrating significant separation between cell groups. **B** Heatmap displaying the expression levels of key marker genes within each cell cluster. The color gradient, ranging from yellow (high expression) to blue (low expression), highlights the significant differences in gene expression between clusters. These expression patterns provide the basis for identifying cell types. **C** Cell type annotation was performed using SingleR, revealing various cell types across the clusters, including T cells, B cells, NK cells, macrophages, monocytes, and epithelial cells. A circular plot shows the distribution of these cell types within the clusters, with different colors representing different cell types. **D** Bar plots showing the proportions of different cell types in tumor and metastatic tissues. The upper panel represents the cell type proportions in tumor tissues, while the lower panel represents the proportions in metastatic tissues. Different colors correspond to different cell types, revealing distinct differences in cell composition between tumor and metastatic tissues

Using UMAP and t-SNE dimensionality reduction analyses on single-cell RNA sequencing data, we identified 25 distinct clusters, each representing a specific cell population (Fig. 2A). The distribution of these clusters in two-dimensional space demonstrates significant separation between cell groups, with different colors representing different clusters. This serves as a foundation for further cell type annotation and functional analysis. A heatmap analysis revealed the expression levels of key genes in each cluster. The color gradient, ranging from red (high expression) to blue (low expression), clearly illustrates the differences in gene expression across clusters, providing strong evidence for the identification and distinction of different cell types (Fig. 2B).Using the SingleR tool for cell type annotation, we further identified the primary cell types in each cluster. The annotation results revealed a variety of immune cell types, including T cells, B cells, NK cells, macrophages, monocytes, and epithelial cells. Heatmaps and circular diagrams detailed the distribution of these cell types within the clusters. The proportions of different cell types across the dataset were visualized using bar plots, highlighting the differences in cell type distribution between tumor and metastatic tissues (Fig. 2C). A clear contrast in the proportions of cell types in tumor and metastatic tissues was evident (Fig. 2D).Functional enrichment analysis revealed significant biological differences among cell types. Functional annotation of key genes within each cluster indicated distinct roles in cell metabolism, immune response, and signal transduction, with color gradients indicating the degree of gene enrichment in each cell type. The functional annotations emphasized the critical roles these genes play in cellular function and biological processes (Fig. 2).In addition, t-tests comparing different cell types between primary tumor and metastatic groups revealed that T cells were more abundant in tumor tissues compared to brain metastases, while epithelial cells were more prevalent in brain metastases than in tumor tissues. Other cell types did not exhibit significant differences between the two groups (Fig. 4).

**Fig. 3:**
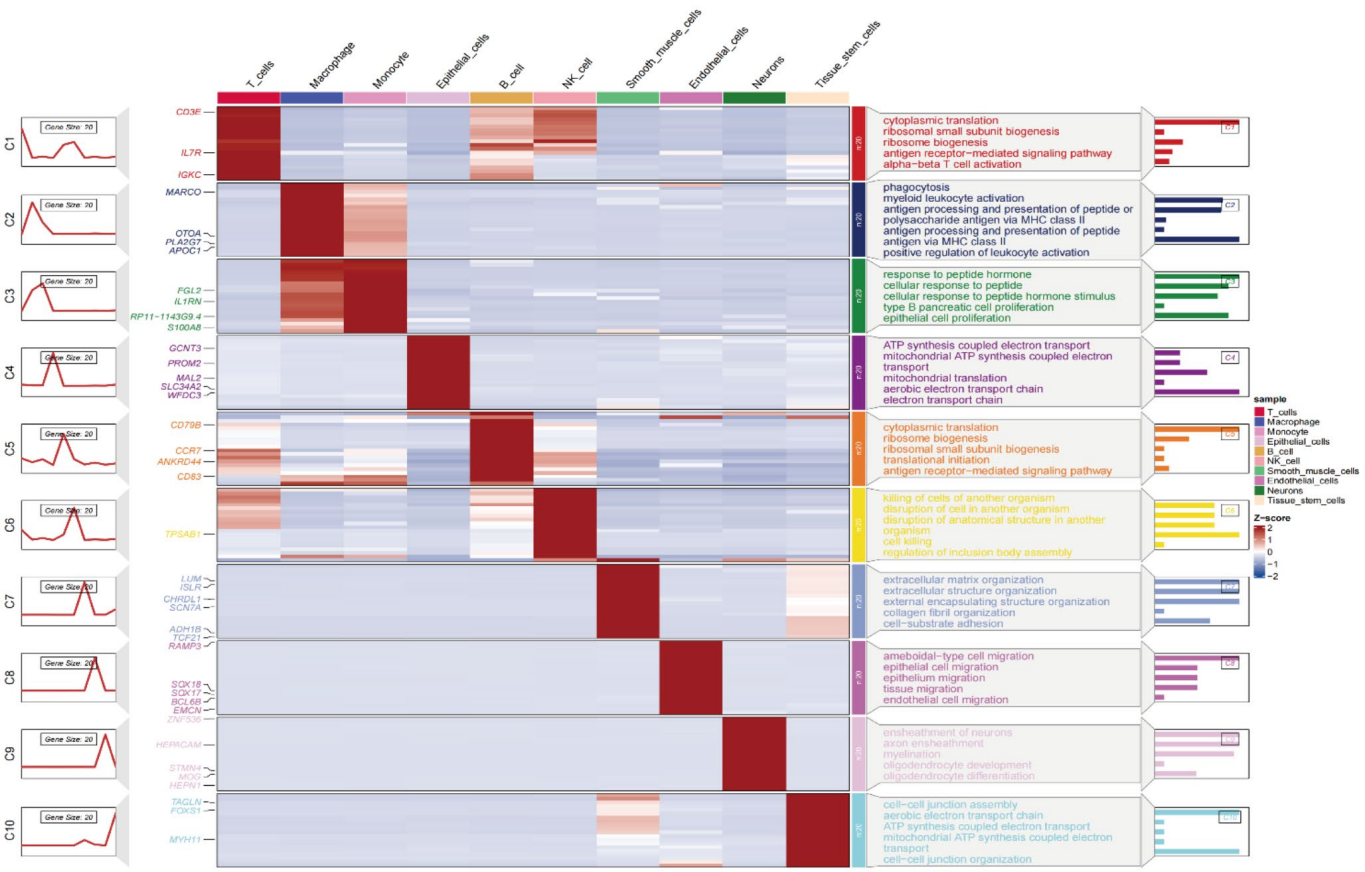
Functional Enrichment Analysis of Different Cell Types The left side displays the expression patterns of each gene cluster (C1–C10), while the heatmap on the right shows the expression levels of these genes across different cell types. Red indicates high expression, and blue indicates low expression. Each gene cluster is associated with specific functions, such as cellular translation, immune response, and signal transduction. The bar chart on the right illustrates the degree of enrichment for these functions in different cell types, helping to uncover the roles of various cell types in biological processes such as metabolism and immunity

**Fig. 4:**
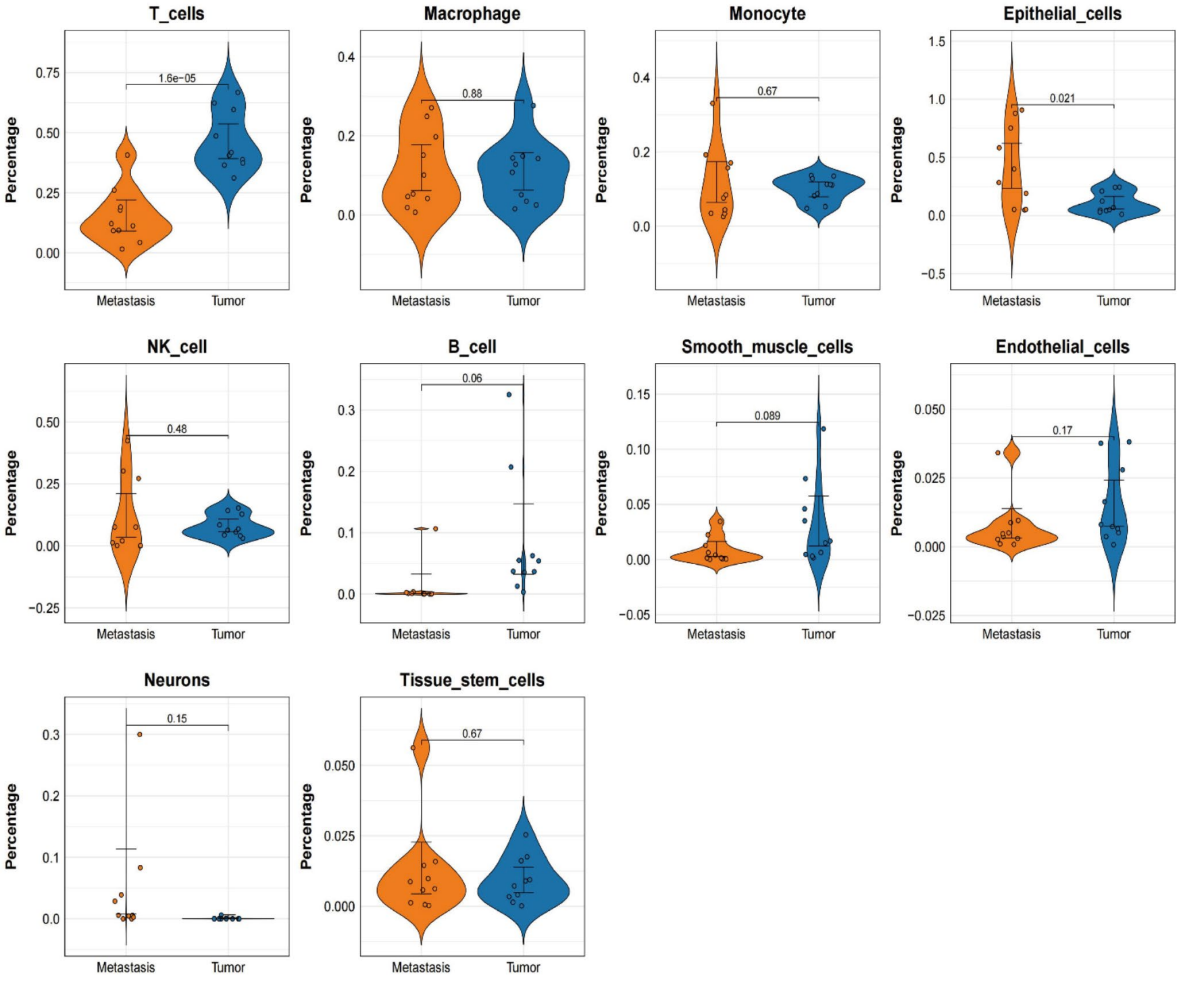
Proportional Differences of Cell Types Between Tumor (Blue) and Metastatic (Orange) Tissues The proportions of different cell types in tumor and metastatic tissues are shown, with T cells being more abundant in tumor tissues compared to metastatic tissues. Conversely, epithelial cells are more prevalent in metastatic tissues than in tumors. No significant differences were observed in the proportions of other cell types, such as macrophages, monocytes, NK cells, and B cells, between the two groups

### Identification of trait-associated genes and cell populations using scPagwas

**Fig. 5:**
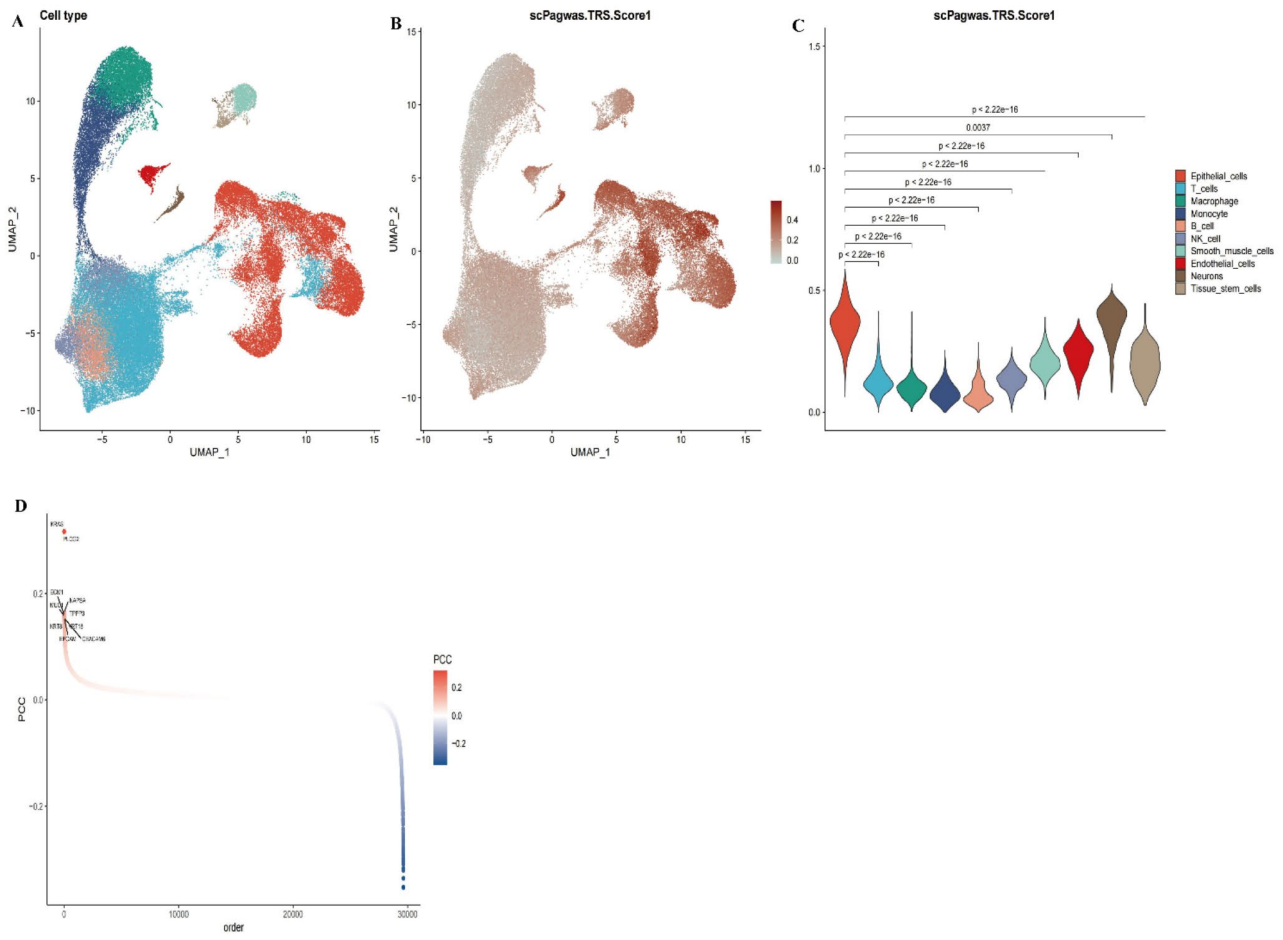
Analysis of Genetic Risk Scores (TRS) in Single-Cell RNA Sequencing Data **A** UMAP plot showing the distribution of different cell types, with distinct colors representing each cell type. **B** UMAP plot displaying the distribution of scPagwas Trait Risk Scores (TRS) across different cell types. The color gradient from light gray to deep red indicates the relative levels of genetic risk scores. **C** Violin plot illustrating the distribution of TRS scores across different cell types, with statistical significance annotated to indicate differences between cell types. (**D**) Scatter plot depicting the correlation between gene heritability and traits. Red indicates a positive correlation, while blue indicates a negative correlation. The top 10 genes significantly associated with traits are highlighted

By performing UMAP dimensionality reduction on single-cell RNA sequencing data (Fig. 5A), we successfully categorized cells into various types, with each type represented by a distinct color. Figure 5B illustrates the distribution of scPagwas Trait Risk Scores (TRS) among these cell populations, where the color gradient ranges from light gray to deep red, indicating variations in genetic risk scores across different cell types. The violin plot in Fig. 5C further quantifies the distribution of TRS scores for each cell type, and statistical significance is annotated to indicate differences between cell types. Finally, the scatter plot in Fig. 5D displays the distribution of genetic heritability and trait association, with colors ranging from blue (negative correlation) to red (positive correlation), highlighting the top 10 genes that show significant associations with traits.

### Differential analysis of TCGA-LUAD

**Fig. 6:**
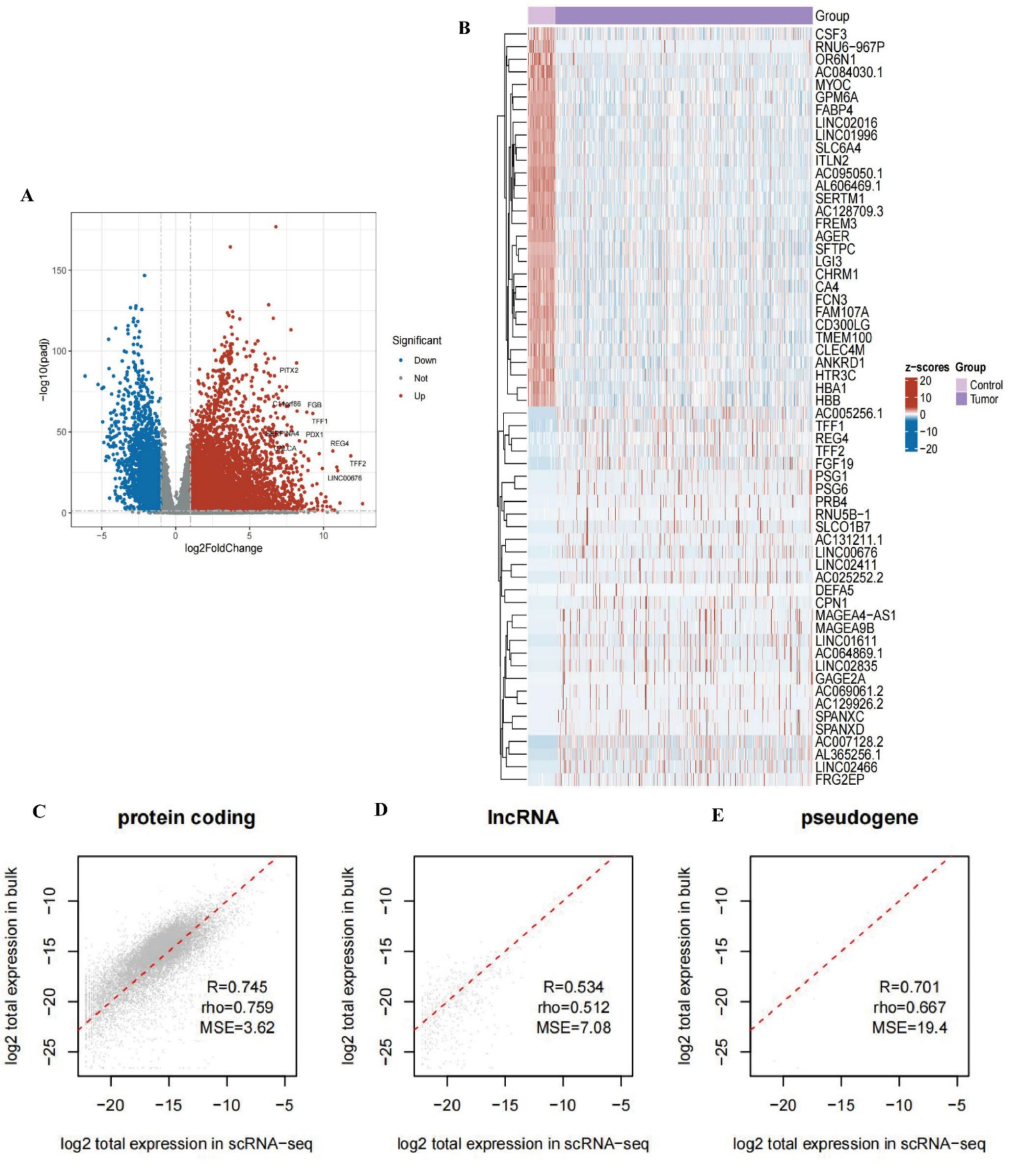
Comparative Analysis of Differential Gene Expression in Bulk and Single-Cell RNA Sequencing Data **A** The volcano plot illustrates the differential gene expression between the tumor and control groups. Red points represent significantly upregulated genes, blue points represent significantly downregulated genes, and gray points indicate genes without significant differences. Key genes, such as PTX2, FGB, and TYRO3, are highlighted. **B** The heatmap displays the expression levels of the top 30 differentially expressed genes between the control and tumor groups. Red indicates high expression, while blue indicates low expression, showing distinct differences in gene expression patterns between the two groups. **C**–**E** Scatter plots compare the consistency of gene expression between single-cell RNA sequencing (scRNA-seq) and bulk RNA sequencing (bulk RNA-seq) data for protein-coding genes **C**, long non-coding RNAs (lncRNAs, **D**), and pseudogenes **E**. Protein-coding genes and pseudogenes exhibit higher consistency between the two sequencing methods, whereas lncRNAs show lower consistency, indicating varying expression characteristics of different gene types across sequencing technologies

To enhance the robustness of our findings, we also performed a differential expression analysis using bulk RNA sequencing data. The volcano plot illustrates the differences in gene expression between the control and tumor groups. The x-axis represents log2FoldChange, and the y-axis represents -log10(padj). Red points indicate upregulated genes, blue points indicate downregulated genes, and gray points represent genes with no significant differences. Key genes, such as PTX2, FGB, and TYRO3, are highlighted in the plot, showing significant upregulation or downregulation (Fig. 6A). On the right, the heatmap displays the top 30 upregulated and downregulated genes with differential expression between the control and tumor groups. Each row represents a gene, and each column represents a sample, with the color gradient ranging from red (high expression) to blue (low expression). Clear distinctions in gene expression patterns between the control and tumor groups are evident (Fig. 6B).

#### Consistency analysis between single-cell RNA sequencing and bulk RNA-seq data

Based on Bayesian deconvolution results, the three scatter plots illustrate the consistency between single-cell RNA sequencing (scRNA-seq) and bulk RNA-seq data in the expression of protein-coding genes, long non-coding RNAs (lncRNAs), and pseudogenes. The scatter plot for protein-coding genes shows high consistency, with a correlation coefficient (R) of 0.745, Spearman’s correlation coefficient (rho) of 0.759, and mean squared error (MSE) of 3.62 (Fig. 6C), indicating strong agreement in gene expression between the two sequencing methods. The scatter plot for lncRNAs demonstrates moderate consistency, with an R value of 0.534, rho of 0.512, and MSE of 7.08, indicating lower consistency in lncRNA expression (Fig. 6D). For pseudogenes, the scatter plot shows relatively high consistency, with an R value of 0.701, rho of 0.667, and MSE of 19.4, though the larger MSE suggests potential bias (Fig. 6E). Overall, these results indicate that scRNA-seq and bulk RNA-seq data exhibit higher consistency in the expression of protein-coding genes and pseudogenes, but lower consistency for lncRNAs, providing important insights into the expression characteristics of different gene types across sequencing technologies.

### Survival analysis of different cell types

The 12 survival curves presented in the figure illustrate the relationship between various cell types (epithelial cells, B cells, endothelial cells, macrophages, monocytes, neurons, NK cells, smooth muscle cells, T cells, and tissue stem cells) and overall survival. Each plot includes survival curves for high-risk and low-risk groups, shown in red and blue, respectively. The analysis results indicate significant differences in survival times between the high-risk and low-risk groups for certain cell types. For example, epithelial cells (survdiff P: 0.026, Cox regression HR: 1.47, 95% CI: 0.6–3.61), B cells (survdiff P: 0.005, Cox regression HR: 0.02, 95% CI: 0.01–0.02), endothelial cells (survdiff P: 0.846, Cox regression HR: 0.23, 95% CI: 0.02–3.39), macrophages (survdiff P: 0.549, Cox regression HR: 0.02, 95% CI: 0.02–1.26), monocytes (survdiff P: 0.947, Cox regression HR: 0.07, 95% CI: 0.16–16.05), neurons (survdiff P: 0.045, Cox regression HR: 0.62, 95% CI: 0.04–9.42), NK cells (survdiff P: 0.264, Cox regression HR: 0.7, 95% CI: 0.22–7.27), smooth muscle cells (survdiff P: 0.702, Cox regression HR: 1.17, 95% CI: 0.3–4.61), T cells (survdiff P: 0.955, Cox regression HR: 0.35, 95% CI: 0.01–11.19), and tissue stem cells (survdiff P: 0.173, Cox regression HR: 1.53, 95% CI: 0.83–5.66) (Figure S3). These findings suggest that the proportion of different cell types is significantly associated with overall survival in patients, offering potential biomarkers and therapeutic targets.

#### Differences in infiltration of various cell types between tumor and adjacent normal tissue

**Fig. 7:**
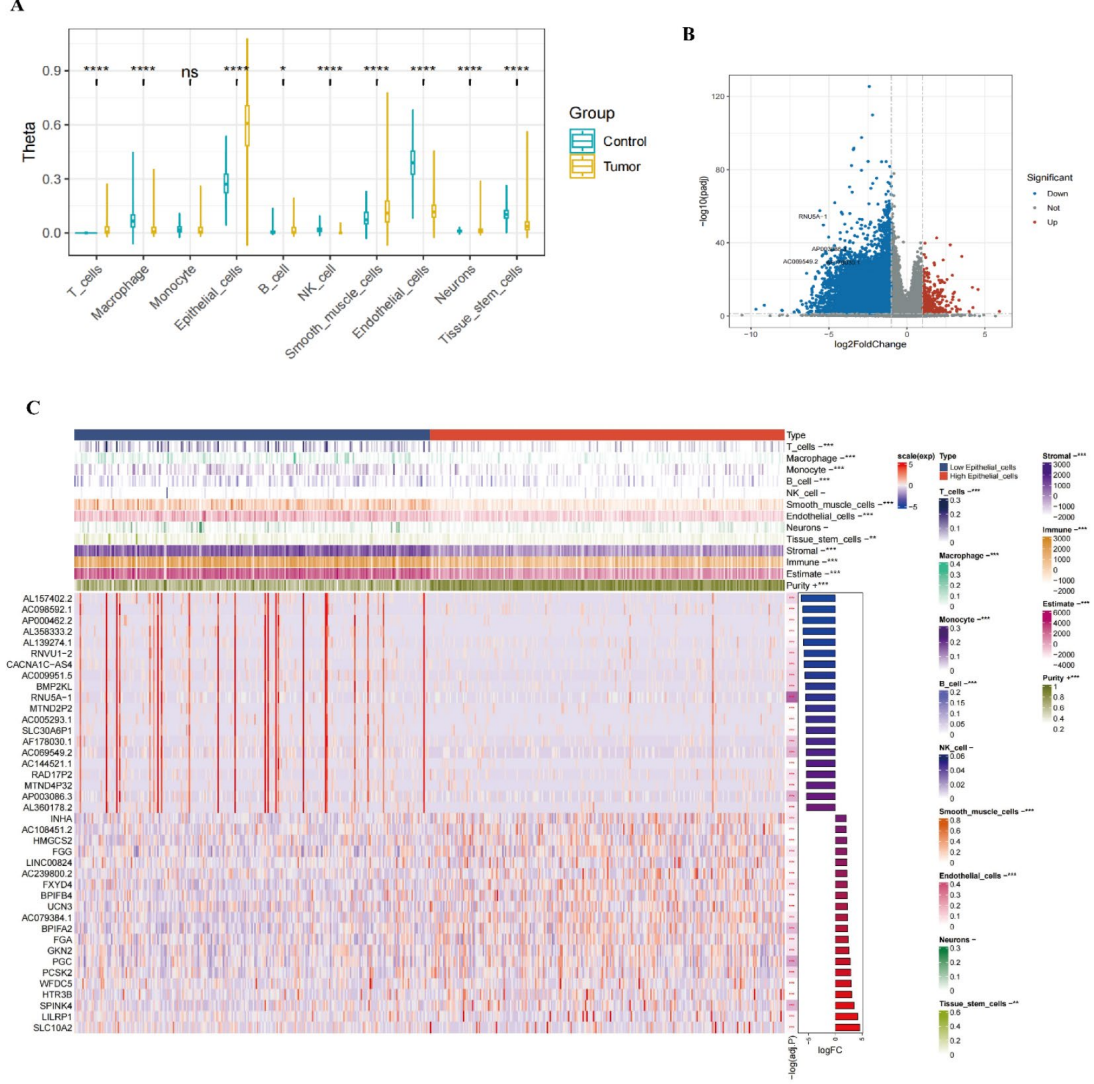
Infiltration Differences of Various Cell Types in Tumor and Adjacent Normal Tissues, and Gene Expression Analysis of Epithelial Cells **A** Box plots display the distribution of Theta values for different cell types in the control group (blue) and tumor group (yellow). The results show significant differences in infiltration levels between the two groups for T cells, macrophages, epithelial cells, B cells, NK cells, smooth muscle cells, endothelial cells, neurons, and tissue stem cells (*p* < 0.05), while monocytes show no significant difference (ns indicates no significant difference). **B** The volcano plot illustrates the gene expression differences between the control group and the high-expression epithelial cell group. Red points represent significantly upregulated genes, and blue points represent significantly downregulated genes. Some key genes, such as RN7J5A-1, AP003046.2, and AC006462.2, show significant upregulation or downregulation in epithelial cells. **C** The heatmap presents the top 20 differentially expressed genes between the control group and the high-expression epithelial cell group. Each row represents a gene, and each column represents a sample. The color gradient from red (high expression) to blue (low expression) reflects changes in gene expression levels. The annotation bar at the top of the heatmap displays different cell types and immune scores (e.g., Stromal, Immune, Estimate, and Purity) with significant differences marked by asterisks (* for *p* < 0.05, ** for *p* < 0.01, *** for *p* < 0.001).

The figure illustrates the distribution of Theta values for different cell types (e.g., T cells, macrophages, monocytes, epithelial cells, B cells, NK cells, smooth muscle cells, endothelial cells, neurons, and tissue stem cells) in the control and tumor groups. Theta values for each cell type are compared between the control and tumor groups, represented by blue and yellow box plots, respectively. The analysis results reveal significant differences in Theta values between the control and tumor groups for certain cell types, such as T cells, macrophages, epithelial cells, B cells, NK cells, smooth muscle cells, endothelial cells, neurons, and tissue stem cells (*p* < 0.05). In contrast, monocytes showed no significant difference (ns) in Theta values between the two groups. These findings suggest differences in the proportions of various cell types between the control and tumor groups, providing important insights into the tumor microenvironment (Fig. 7A).

#### Differential analysis of epithelial cell subgroups

Previous evidence has demonstrated that epithelial cells play a crucial role in the development and progression of lung cancer. In this next stage of analysis, we focus on epithelial cells, using a volcano plot to visualize gene expression differences between the control group and the high-expression epithelial cell group. The x-axis represents log2FoldChange, while the y-axis represents -log10(padj). Red points indicate significantly upregulated genes, blue points indicate significantly downregulated genes, and gray points represent genes with no significant differences. Key genes, such as RN7J5A-1, AP003046.2, and AC006462.2, show significant upregulation or downregulation (Fig. 7B).The heatmap illustrates the expression differences of the top 20 significantly upregulated and downregulated genes in both the control and high-expression epithelial cell groups. Each row represents a gene, and each column represents a sample, with colors ranging from red (high expression) to blue (low expression). The annotation bar at the top indicates information such as different cell types and immune scores, with color coding to differentiate between groups. In the volcano plot, the log2FoldChange on the x-axis shows the magnitude of gene expression changes, with values farther from zero representing greater changes. The -log10(padj) on the y-axis indicates the level of statistical significance, with higher values representing stronger significance. Red and blue points represent upregulated and downregulated genes, respectively, highlighting the differential expression between the two groups.In the heatmap, each row represents a differentially expressed gene, and each column represents a sample. The color gradient from red (high expression) to blue (low expression) illustrates the gene expression levels across samples. The top annotation bar includes various cell types (e.g., T cells, macrophages, monocytes, epithelial cells, B cells, NK cells, smooth muscle cells, endothelial cells, neurons, and tissue stem cells) and immune scores (e.g., Stromal, Immune, Estimate, and Purity). Significant differences between the control and high-expression epithelial cell groups are indicated by asterisks (* for *p* < 0.05, ** for *p* < 0.01, *** for *p* < 0.001). The Stromal (stromal score), Immune (immune score), Estimate (composite score), and Purity (purity score) represent the relative abundance of different components in the tumor microenvironment, with significant differences also marked by asterisks.

Through this analysis, we identified genes that are significantly upregulated or downregulated in the high-expression epithelial cell group and visualized their expression patterns in the control and high-expression groups. The volcano plot and heatmap provide clear visualizations of differential gene expression between the two groups, offering valuable insights into the gene expression characteristics within the tumor microenvironment (Fig. 7C). 

#### Clinical trait analysis of epithelial cell subgroups

The figure illustrates the distribution of epithelial cells across different clinical characteristics (T stage, N stage, M stage, gender, age, and clinical stage). The analysis shows a significant difference in epithelial cell content between the T1 and T3 groups of the T stage (*p* < 0.05) (Supplementary Fig. 4A). However, no significant differences were observed in the relative abundance of epithelial cells across groups for N stage, M stage, gender, age, and clinical stage. This indicates that, aside from the T stage, the relative abundance of epithelial cells does not vary significantly across other clinical characteristics. These findings provide a basis for further exploration of the specific role of epithelial cells in tumor progression (Supplementary Fig. 4B, C, D, E, F).

### Relationship between epithelial cells and immune-related gene sets

In this study, we integrated single-cell RNA sequencing data, GWAS, Bayesian deconvolution, and machine learning techniques to analyze the expression patterns of immune-related genes in epithelial cells with different expression levels.

**Fig. 8:**
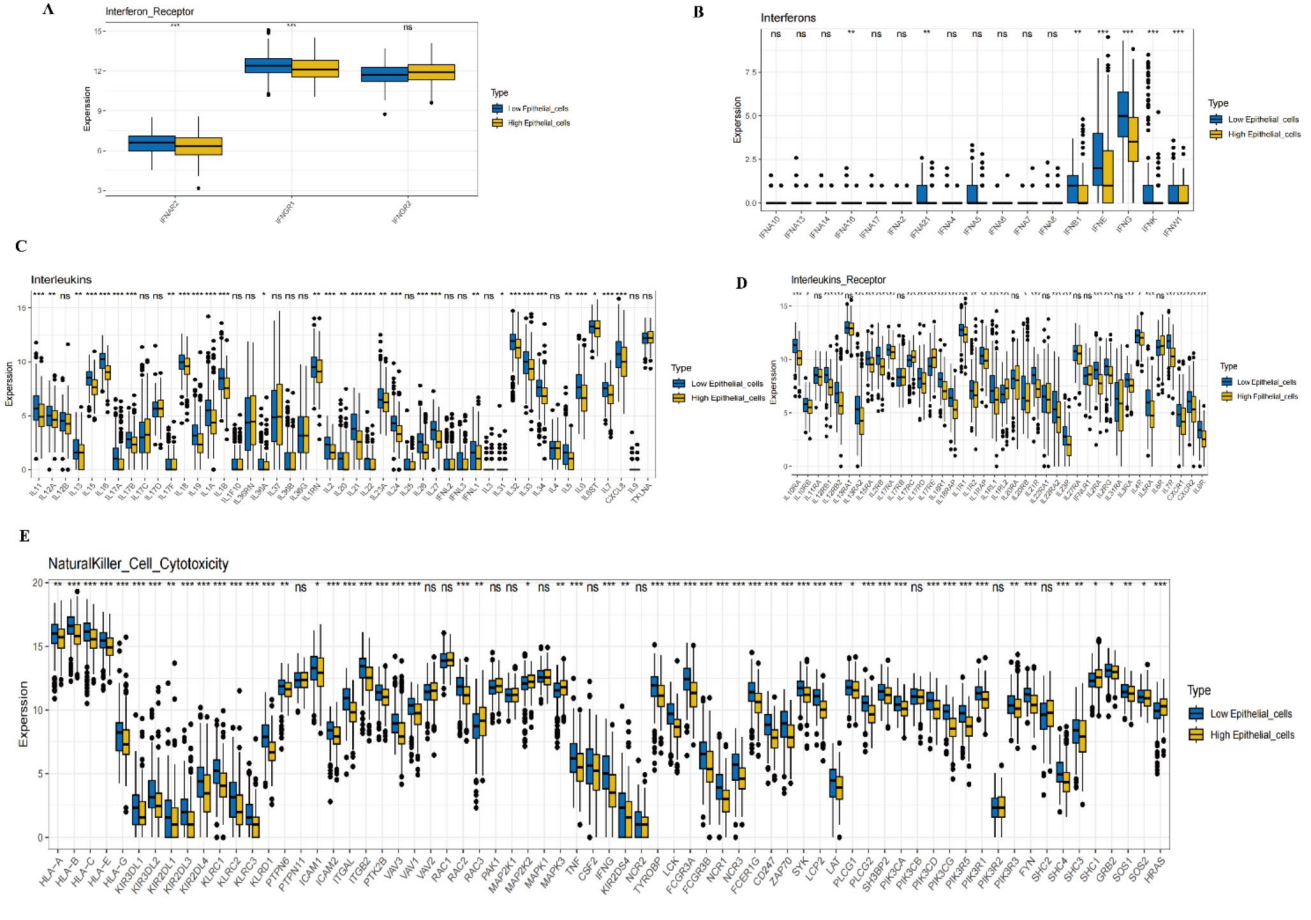
Expression Relationships Between Epithelial Cells and Immune-Related Gene Sets **A** Interferon Receptor Genes: This panel shows the expression levels of interferon receptor genes (e.g., IFNAR1, IFNAR2, IFNGR1, IFNGR2) in high-expression and low-expression epithelial cells. The expression of interferon receptor genes is significantly higher in high-expression epithelial cells compared to the low-expression group, displaying highly significant differences (*p* < 0.001). **B** Interferon Genes: This panel presents the expression of various interferon genes in epithelial cells. Most interferon genes show no significant difference between high-expression and low-expression cells (ns indicates no significance), though a few genes exhibit slight upregulation in high-expression epithelial cells. **C** Interleukin Genes: This panel shows the expression levels of multiple interleukin genes (e.g., IL1, IL2, IL6, IL10), with some genes significantly upregulated in high-expression epithelial cells. **D** Interleukin Receptor Genes: The expression levels of interleukin receptor genes are significantly higher in high-expression epithelial cells compared to low-expression cells. **E** Natural Killer Cell Cytotoxicity Genes: Genes associated with natural killer cell cytotoxicity (e.g., KLRD1, NKG7, GZMA, GZMB) are expressed at significantly higher levels in high-expression epithelial cells compared to the low-expression group

Interferon Receptors: This figure shows the expression levels of interferon receptor genes (IFNAR1, IFNAR2, IFNGR1, IFNGR2) in high-expression and low-expression epithelial cells. The results indicate that all receptor genes are significantly more highly expressed in high-expression epithelial cells compared to low-expression cells. This suggests that interferon receptors may play distinct functional roles in epithelial cells with varying expression levels (Fig. 8A).Interferons: This figure displays the expression distribution of interferon family genes (e.g., IFNA, IFNB, IFNG) across different epithelial cell types. Most genes show no significant difference (“ns”), although a few genes exhibit slightly higher expression in high-expression cells. This implies that the role of interferons may not differ significantly between these two cell types (Fig. 8B).Interleukins: The figure presents the expression levels of several interleukin genes (e.g., IL1, IL2, IL6, IL10) in the two cell types. Significant differences are marked with asterisks, showing that some interleukins are significantly upregulated in high-expression epithelial cells, suggesting that these cells may be more actively involved in specific immune responses (Fig. 8C).Interleukin Receptors: This figure illustrates the expression differences of interleukin receptor genes, with most receptors showing higher expression in high-expression cells. This suggests that these receptors may play an important role in immune signaling within epithelial cells, influencing their immune responses (Fig. 8D).Natural Killer Cell Cytotoxicity: The figure shows the expression levels of genes associated with natural killer cell cytotoxicity (e.g., KLRD1, NKG7, GZMA, GZMB), revealing that these genes are significantly more highly expressed in high-expression epithelial cells compared to low-expression cells. This suggests that these genes may play a key role in regulating the immune characteristics of epithelial cells (Fig. 8E).

### Immunotherapy analysis of epithelial cells

We observed significant differences in several immune-related markers between the high- and low-expression epithelial cell groups. Notably, CD8 and PD-L1 (CD274) were significantly upregulated in the high-expression group, suggesting enhanced immune cell activity and infiltration in these samples. Additionally, the expression of immune inhibitory markers CTLA-4 and the metabolic enzyme IDO1 was also significantly higher in the high-expression group, indicating a more complex tumor microenvironment with activated immune evasion mechanisms.Regarding immunotherapy-related biomarkers, the significant upregulation of IFNG suggests a strong Th1-type immune response, which is associated with a favorable potential for immunotherapy response. We also observed a notable increase in tumor mutational burden (TMB) and microsatellite instability (MSI) score in the high-expression group, further supporting the idea that these samples may have enhanced responsiveness to immune checkpoint inhibitors.These findings provide valuable biomarkers and potential therapeutic targets for future immunotherapies in tumors with high epithelial cell expression (Fig. S5).

## Drug response prediction for epithelial cells

This figure presents the differences in drug response between the two sample groups (“Low Epithelial_cells” and “High Epithelial_cells”). The box plots show the distribution of drug response levels across different sample groups. The figure highlights that, for certain drugs, the “High Epithelial_cells” group exhibits a significantly higher response compared to the “Low Epithelial_cells” group, suggesting that these drugs may be more effective for patients with high epithelial cell expression. This information could be highly valuable in developing personalized treatment strategies(Figure S6).

### Single nucleotide variant (SNV) analysis in epithelial cells

**Fig. 9:**
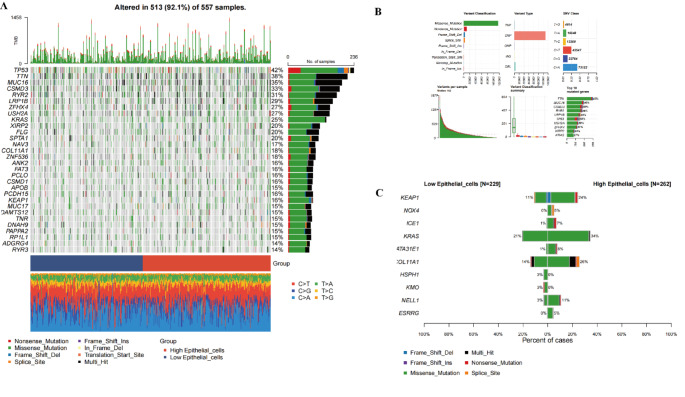
SNV (Single Nucleotide Variant) Analysis in Epithelial Cell Populations **A** Mutation Frequency Heatmap: This heatmap illustrates the mutation frequencies across 557 samples, with 513 samples (92.1%) exhibiting genetic mutations. The colors represent different types of mutations, including missense mutations, nonsense mutations, frameshift insertions, frameshift deletions, and splice site variants. The bar chart at the top displays the mutation burden for each sample, with some showing a significantly higher mutation load. **B** Analysis of Mutation Types and Frequencies: The bar chart in the top right shows the frequencies of various mutation types, including single nucleotide polymorphisms (SNPs), deletions (DEL), and insertions (INS). Below, the mutation rates of key genes, such as TP53 and TTN, are presented, indicating that these genes have high mutation frequencies across multiple samples, which suggests they may play a crucial role in tumor progression. **C** Comparison of Mutation Frequencies in Low and High Epithelial Cell Populations: The bar chart compares the mutation frequencies of key genes in the low-epithelial cell group (*N* = 229) and the high-epithelial cell group (*N* = 262). The analysis reveals that genes such as KRAS and KEAP1 have significantly higher mutation rates in the high-epithelial cell group, suggesting that these mutations may be closely related to epithelial cell states and could impact tumor development and progression

The heatmap and bar chart on the left provide a comprehensive view of mutation frequencies, showing that 513 out of 557 samples (92.1%) exhibit genetic mutations. The color coding represents various types of mutations, including missense mutations, nonsense mutations, frameshift insertions, frameshift deletions, and splice site variants. The bar chart at the top displays the total mutation burden for each sample, revealing an abnormally high number of mutations in some samples (Fig. 9A).The bar chart at the top right details the frequencies of different mutation types, illustrating the distribution of single nucleotide polymorphisms (SNPs), deletions (DEL), and insertions (INS). Additionally, it shows the mutation rates of genes such as TP53 and TTN, which may play critical roles in tumor development due to their frequent mutations across multiple samples (Fig. 9B).The two bar charts at the bottom compare the mutation frequencies in low-epithelial and high-epithelial cell states. By contrasting the mutations in key tumor suppressor genes and oncogenes, such as KRAS and KEAP1, across different cell phenotypes, we gain a deeper understanding of how these mutations contribute to tumor biology and their potential impact on cancer progression (Fig. 9C).

### WGCNA analysis of convolutional cells

**Fig. 10:**
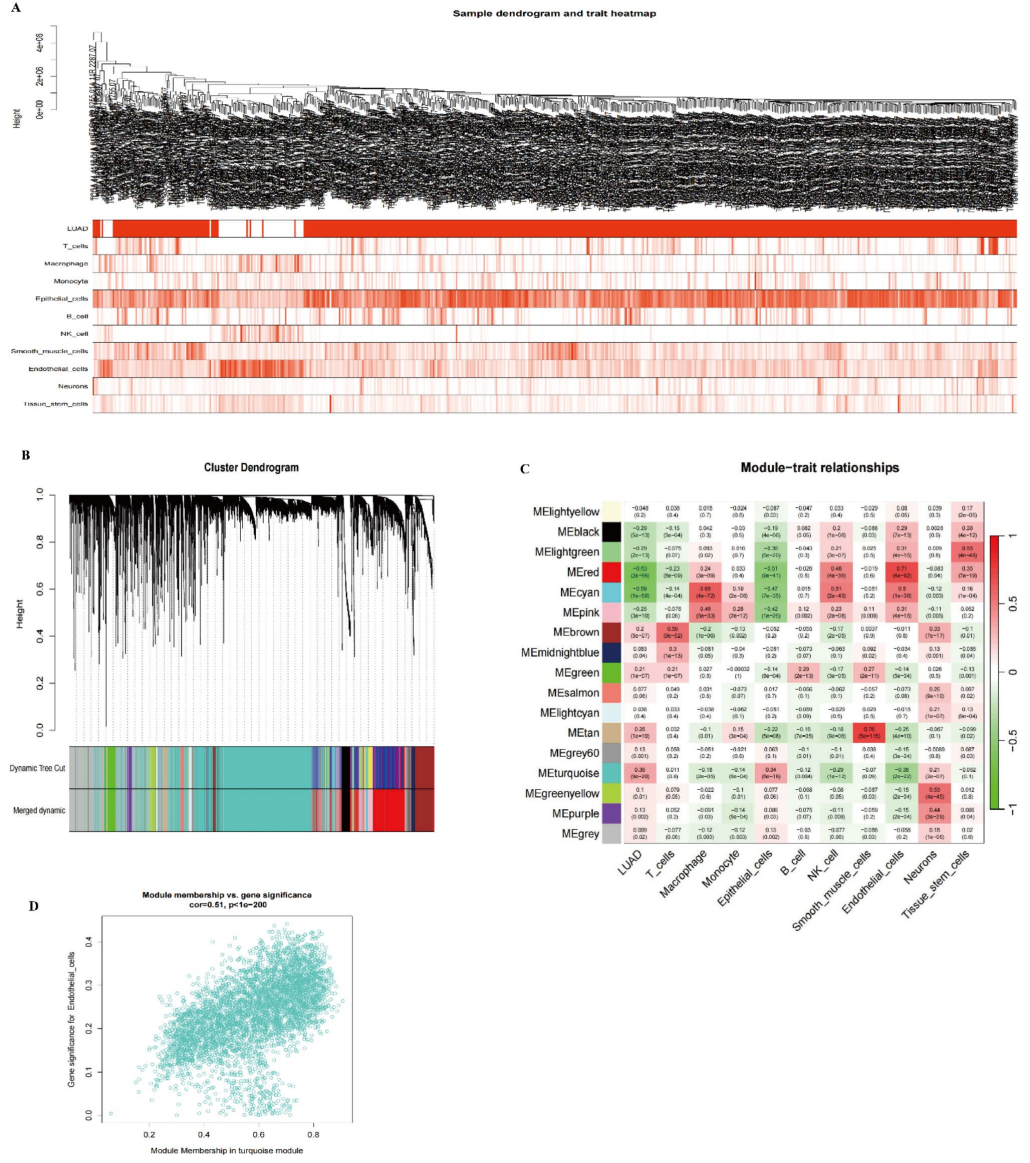
WGCNA (Weighted Gene Co-expression Network Analysis) of Convolutional Cells **A** Sample Clustering Dendrogram and Trait Heatmap: This panel displays the clustering results of LUAD (lung adenocarcinoma) samples along with the corresponding trait heatmap, used to verify the quality and consistency of the samples. The heatmap illustrates the infiltration levels of different immune cell types (e.g., T cells, macrophages, NK cells) across various samples. **B** Gene Clustering Dendrogram: This dendrogram shows the clustering results of genes, identifying multiple co-expression gene modules. Different colors represent distinct gene modules, with the green module showing a significant correlation with endothelial cell infiltration. **C** Module-Trait Association Heatmap: This heatmap presents the correlations between different gene modules and immune cell infiltration characteristics. The green module is significantly associated with immune cell types such as endothelial cells, T cells, and macrophages. **D** Module Membership vs. Gene Significance Plot: This plot illustrates the relationship between module membership and gene significance within the green module, particularly highlighting its association with endothelial cell infiltration

In this study, we utilized Weighted Gene Co-expression Network Analysis (WGCNA) to identify gene co-expression modules in lung adenocarcinoma (LUAD) samples and explored the relationship between these modules and immune infiltration characteristics. First, we verified the quality and consistency of the samples through a sample clustering dendrogram and trait heatmap (Fig. 10A), ensuring the reliability of the analysis results. Next, by constructing a gene clustering dendrogram (Fig. 10B), we identified several biologically meaningful gene modules. Notably, the green module showed a significant positive correlation with endothelial cell infiltration levels, suggesting that the genes within this module may play a key role in regulating the tumor microenvironment.The module-trait association heatmap (Fig. 10C) further revealed the correlations between various modules and immune cell types, underscoring the green module’s critical role in LUAD. Additionally, through the module membership versus gene significance plot (Fig. 10D), we identified key genes within the module that have both high significance and strong module membership.

#### Screening of core differential genes in epithelial cells

**Fig. 11:**
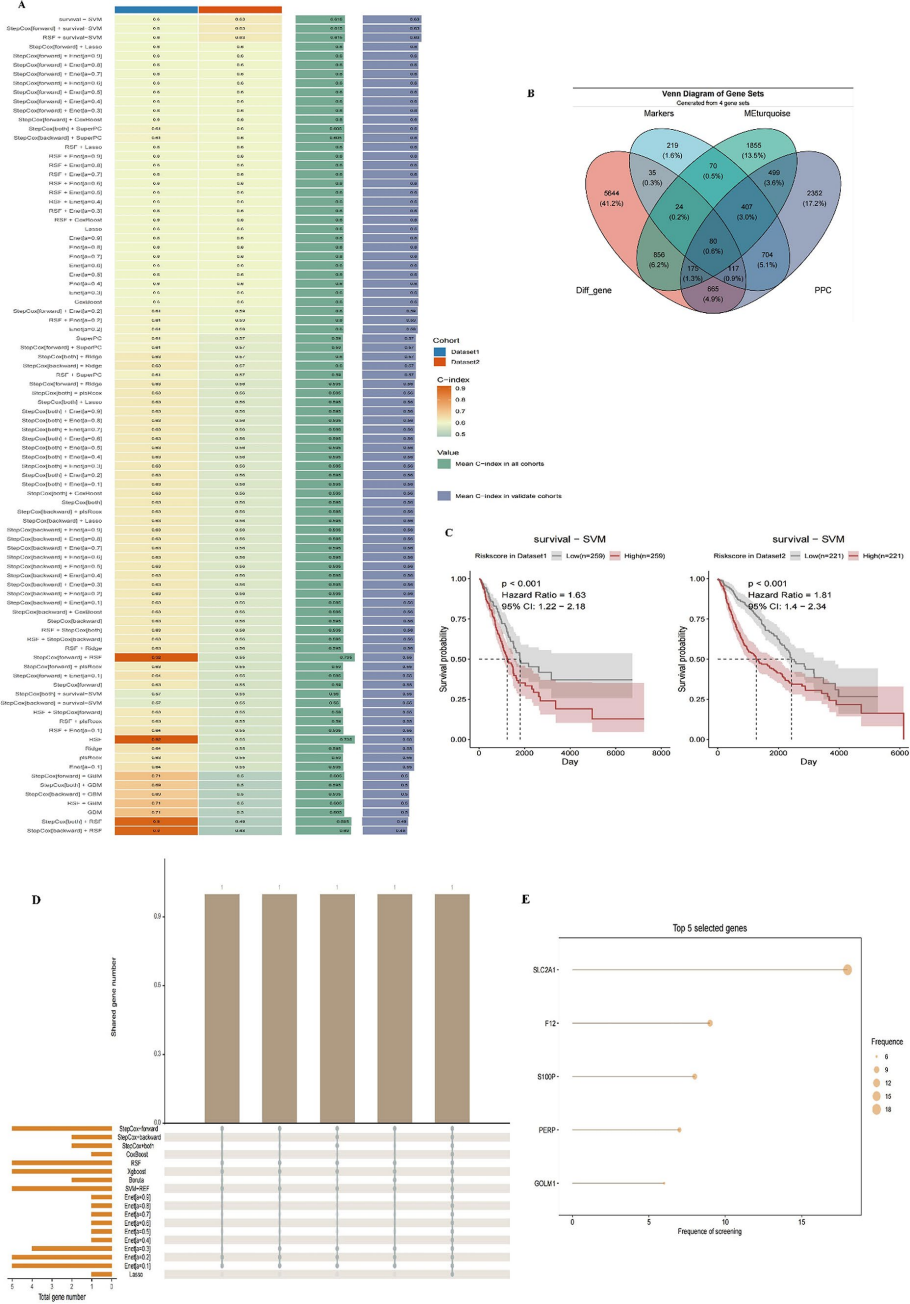

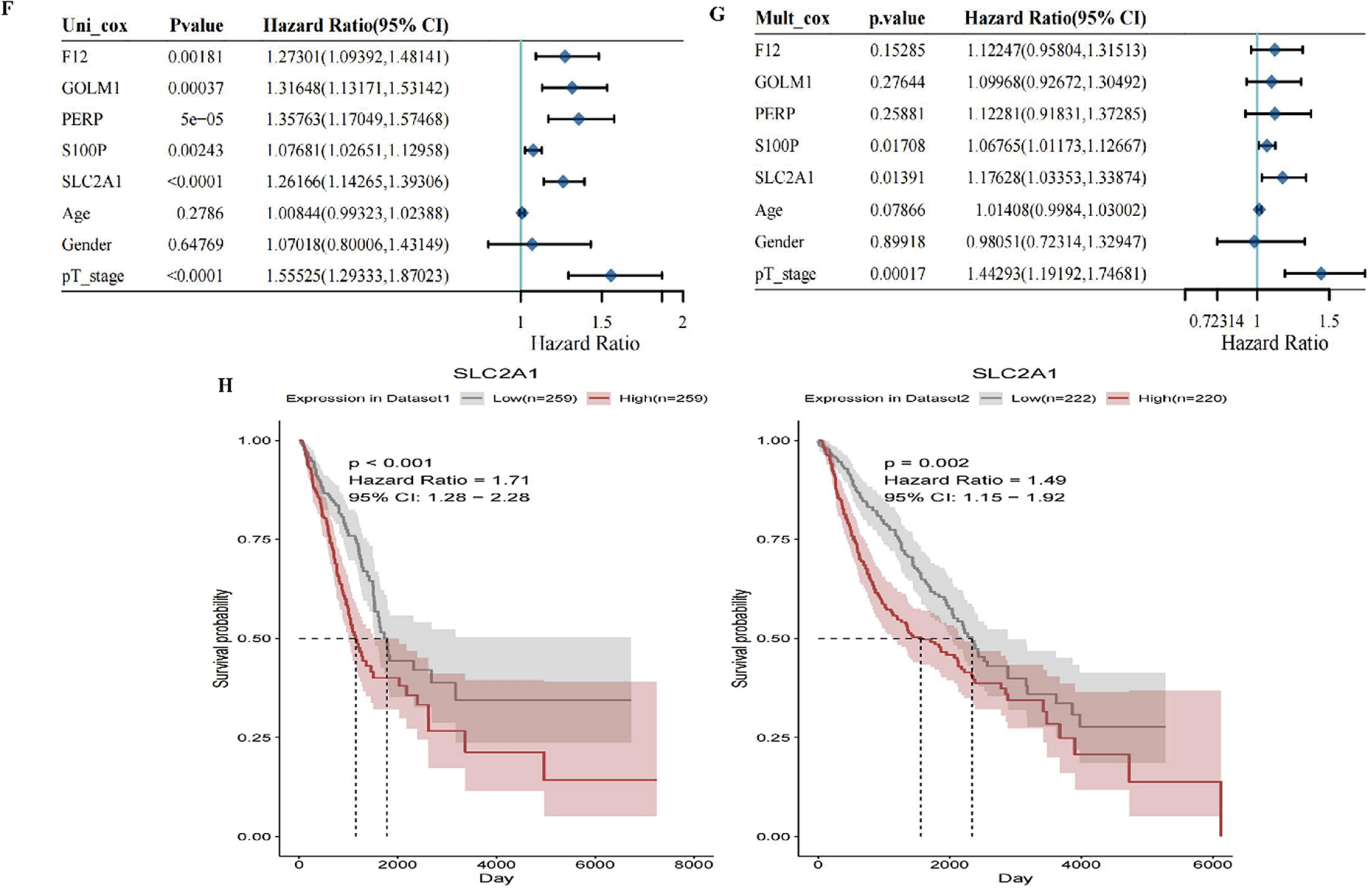
Screening of Core Differential Genes in Epithelial Cells and Survival Analysis **A** Heatmap of Machine Learning Model Performance: This heatmap illustrates the performance of different machine learning models across two datasets (Dataset1 and Dataset2), with color intensity representing C-index values; higher C-index values indicate better predictive performance. **B** Venn Diagram: This diagram displays the overlap among four gene sets: Markers, MEturquoise, Diff_gene, and PPC. The final intersection includes 80 core genes. **C** Survival Curve Plot: Based on the survival - SVM model, patients are stratified into high-risk and low-risk groups. The left plot corresponds to Dataset1, while the right plot corresponds to Dataset2, showing that the survival probability of high-risk patients is significantly lower than that of low-risk patients. **D** Bar Chart of Gene Selection Frequency: This chart presents the frequency of the top five genes identified during multiple screenings. **E** Univariate and Multivariate COX Regression Analysis: Univariate analysis reveals that the genes F12, GOLM1, PERP, S100P, and SLC2A1 are significantly associated with poor survival. In multivariate analysis, S100P, SLC2A1, and pT_stage remain significant, indicating their status as independent prognostic factors. **F** Survival Analysis of SLC2A1 Gene: This section showcases the survival curves of the SLC2A1 gene across Dataset1 and Dataset2. **G** Multivariate Cox Regression Analysis: The results of the multivariate Cox regression are depicted, focusing on the impact of F12, GOLM1, PERP, S100P, SLC2A1, and clinical characteristics (age, gender, pT_stage) on patient survival rates. **H** Survival Curve Analysis of SLC2A1 Gene in Dataset1 and Dataset2: This plot displays the survival curves for patients grouped by high and low expression levels of the SLC2A1 gene

The Venn diagram (Fig. 11B) illustrates the overlap among four gene sets: Markers, MEturquoise, Diff_gene, and PPC, with a final intersection comprising 80 common genes. Each gene set plays a distinct role in biological processes; Markers represent specific biomarker genes, MEturquoise indicates significant modules identified through WGCNA analysis, Diff_gene contains genes with significant differential expression, and PPC is associated with specific pathological processes.

#### Machine learning screening of key genes through univariate and multivariate COX regression analysis

In this study, we employed various machine learning methods to construct models for cancer prognosis prediction, validating them across different datasets. The heatmap (Fig. 11A) displays the performance of different models on two datasets (Dataset1 and Dataset2), with color intensity reflecting the C-index values; higher C-index values indicate better predictive performance. The survival - SVM model consistently demonstrated high C-index values across both datasets, indicating strong predictive capabilities. Survival curves (Fig. 11C) based on the survival - SVM model’s risk scores stratified patients into high-risk and low-risk groups, with clearly separated curves indicating the model’s effectiveness in distinguishing survival rates among different risk groups. The left survival curve corresponds to Dataset1, while the right corresponds to Dataset2. The gene screening results highlight the top five genes with the highest selection frequency during the screening process (Fig. 11D). These genes, considered core to patient survival, include SLC2A1, F12, S100P, PERP, and GOLM1, with dot size representing gene appearance frequency across multiple screenings (Fig. 11F). Univariate analysis results (Fig. 11F) indicate that F12 (HR = 1.27, *p* = 0.00181), GOLM1 (HR = 1.51, *p* = 0.00037), PERP (HR = 1.35, *p* = 5e-05), S100P (HR = 1.08, *p* = 0.00243), SLC2A1 (HR = 1.26, *p* < 0.0001), and pT_stage (HR = 1.55, *p* < 0.0001) are significantly associated with poor survival. Age and gender did not show significance. Multivariate analysis (Fig. 11G) further confirms that S100P (HR = 1.07, *p* = 0.01708), SLC2A1 (HR = 1.18, *p* = 0.01391), and pT_stage (HR = 1.44, *p* = 0.00017) remain significant, indicating that these genes and clinical features are independent prognostic factors. Notably, SLC2A1 exhibited significance in both univariate and multivariate analyses, suggesting it as the most promising prognostic biomarker. Figure 11 H presents the survival curves for the SLC2A1 gene in Dataset1 and Dataset2, revealing that patients with high SLC2A1 expression have significantly lower survival rates compared to those with low expression (Dataset1: HR = 1.71, *p* < 0.001; Dataset2: HR = 1.49, *p* = 0.002). This indicates that high SLC2A1 expression may correlate with poor prognosis, highlighting its potential as a biomarker for personalized prognosis assessment in cancer treatment. These findings provide critical information for biomarkers in personalized treatment strategies for cancer patients.

## Expression pattern of SLC2A1 in epithelial cells

**Fig. 12:**
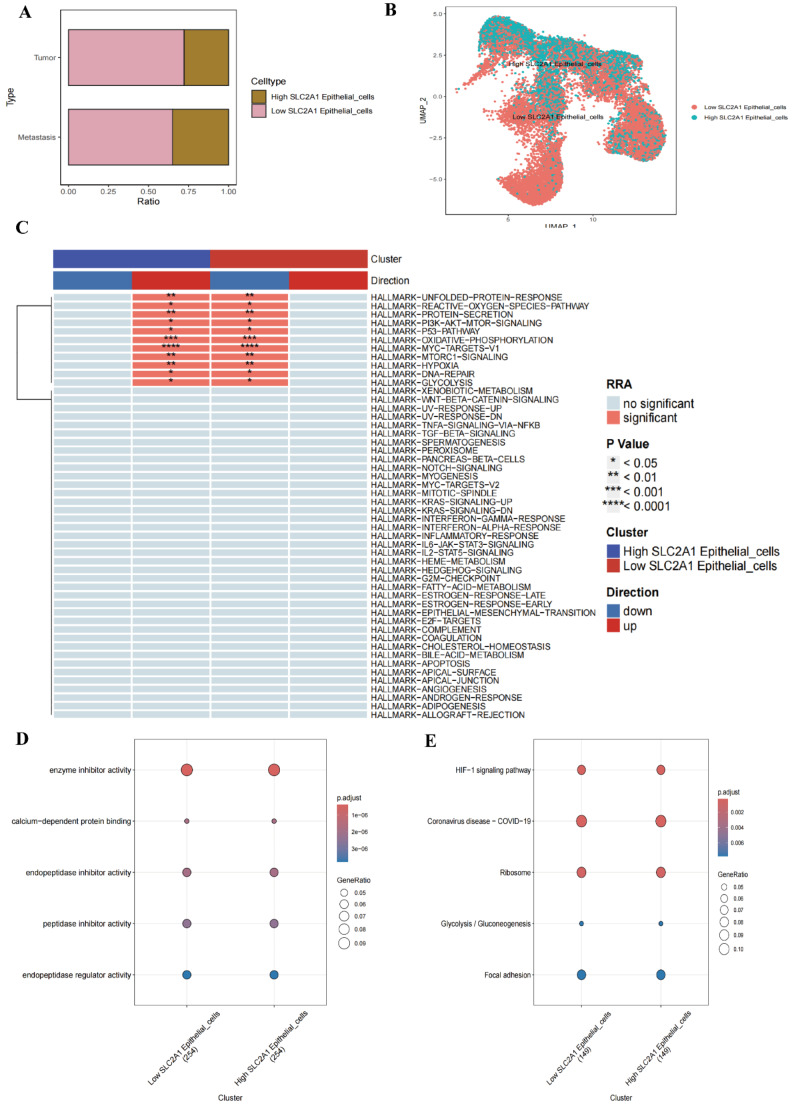
Expression Pattern and Functional Analysis of SLC2A1 in Epithelial Cells **A** Cell Population Proportion Analysis: This analysis illustrates the differences in the proportions of epithelial cells with high and low SLC2A1 expression in primary versus metastatic tumors. **B** UMAP Analysis: UMAP visualizations show a clear separation of epithelial cell populations with high and low SLC2A1 expression in feature space. **C** Gene Set Enrichment Analysis: Differential gene analysis and gene set enrichment analysis indicate that cells with high SLC2A1 expression are significantly enriched in signaling pathways related to oxidative stress response, apoptosis, metabolism, and immune evasion. **D** GO Enrichment Analysis: GO enrichment analysis based on differentially expressed genes associated with high and low SLC2A1 expression reveals significant enrichment in functions such as enzyme inhibitory activity and endonuclease activity among the high SLC2A1 expressing cell population. **E** KEGG Pathway Enrichment Analysis: KEGG analysis demonstrates that cells with high SLC2A1 expression are enriched in multiple signaling pathways, including the HIF-1 signaling pathway, glycolysis/gluconeogenesis, and ribosome pathways

In our study, analysis of the expression levels of the SLC2A1 gene in epithelial cells revealed that high SLC2A1 expression is significantly associated with the enrichment of specific cell subpopulations. This suggests that SLC2A1 may play a crucial role in various tumor cell types or cellular states, particularly concerning tumor aggressiveness and metastatic potential. Through population proportion analysis, we observed a significant difference in the proportions of epithelial cells expressing high versus low levels of SLC2A1 in primary versus metastatic tumors. Specifically, the proportion of SLC2A1 high-expressing cells was greater in metastatic tumors, reflecting the gene’s important role in the metastatic process (Fig. 12A). UMAP and t-SNE analyses further supported these findings, clearly demonstrating the separation of cell populations with high and low SLC2A1 expression in feature space. This visual separation validates that the expression level of SLC2A1 can effectively distinguish between different cell populations, suggesting its potential role in cell differentiation and tumor heterogeneity. Differential gene analysis of high versus low SLC2A1 expression groups identified a set of significant differentially expressed genes associated with metabolism, the cell cycle, and signaling pathways (Fig. 12B). GO and KEGG pathway enrichment analyses further confirmed that key biological processes involved in high SLC2A1 expressing cells may be closely linked to tumor energy metabolism and cell proliferation (Fig. 12D, E). Utilizing the irGSEA method for gene set enrichment analysis, we found that cells with high SLC2A1 expression are significantly enriched in signaling pathways related to oxidative stress response, apoptosis, and immune evasion (Fig. 12C). This indicates that SLC2A1 not only plays a role in tumor cell growth and metastasis but may also influence the tumor microenvironment and its interaction with the immune system.

## Cell trajectory analysis

**Fig. 13:**
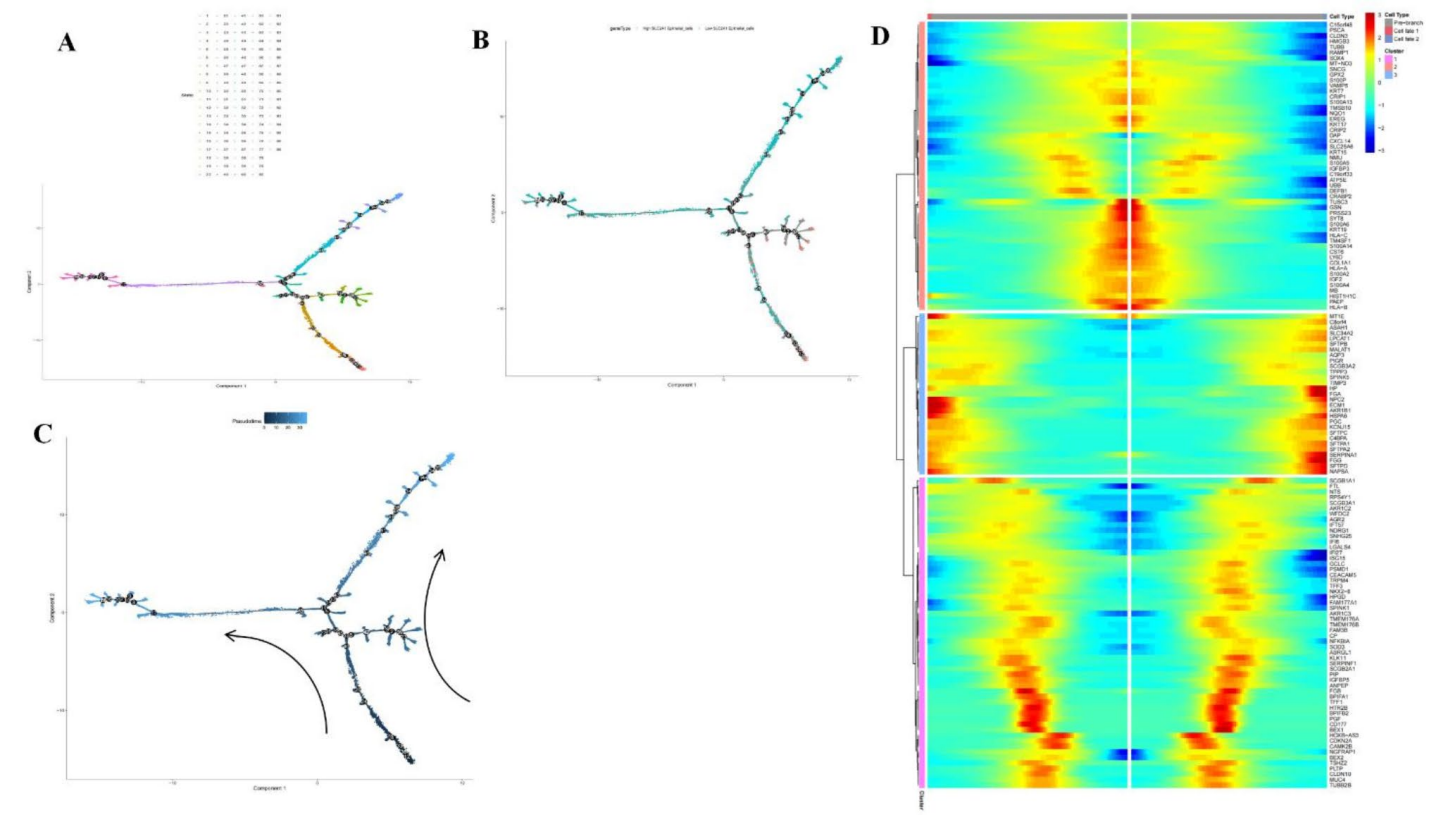
Cell Trajectory Analysis of Epithelial Cells with High and Low SLC2A1 Expression Figure**A** illustrates the pseudotime trajectory of epithelial cells, depicting their differentiation along various pathways from an initial state to multiple branching points, which represent critical decision nodes in cell differentiation. Figure** B** presents the trajectories of high (red) and low (blue) SLC2A1 expressing cells, revealing that cells with high SLC2A1 expression tend to cluster along specific branches, suggesting a potential drive towards particular differentiation directions. Figure** C** further demonstrates the direction of cell migration, with arrows indicating the primary pathways of movement. Figure ** D** features a gene expression heatmap based on BEAM analysis, showcasing significant changes in gene expression across different differentiation pathways, highlighting the potential roles of key regulatory genes in cell differentiation and tumor progression

In our study, we employed Monocle to perform cell trajectory analysis on epithelial cells with high and low SLC2A1 expression, successfully reconstructing their pseudotime pathways. By identifying the differentiation states and trajectories of these cells (Fig. 13A and B), we observed the potential developmental and differentiation processes over time. This analysis not only enhances our understanding of the dynamic changes in cells with different expression levels but also reveals the potential role of SLC2A1 in cellular differentiation.Figure A presents the pseudotime trajectory of the cells, illustrating their dynamic migration from an initial state to multiple branching points, which represent critical decision points in cell differentiation. Figure B further refines the trajectories of high and low SLC2A1 expressing cells, showing that cells with high SLC2A1 expression primarily cluster at specific branches, suggesting their importance in directing cells towards particular differentiation pathways. Figure 13 C utilizes the pseudotime path to demonstrate the dynamic migration of cells over time, with arrows indicating important directions of cell migration. Employing Branch Expression Analysis Modeling (BEAM) (Fig. 13D), we confirmed significant changes in gene expression at these branching points and their associated functions. The heatmap analysis of gene expression highlights clusters of genes that exhibit significant changes during the branching process, emphasizing the expression differences of specific genes along various differentiation pathways. By categorizing these genes into distinct clusters, we identified several key regulatory genes that may play critical roles in SLC2A1-mediated cell migration and functional changes. Certain upregulated genes in cells with high SLC2A1 expression underscore its pivotal role in tumor progression and alterations in cellular functions.

### Comparative analysis of cell communication

**Fig. 14:**
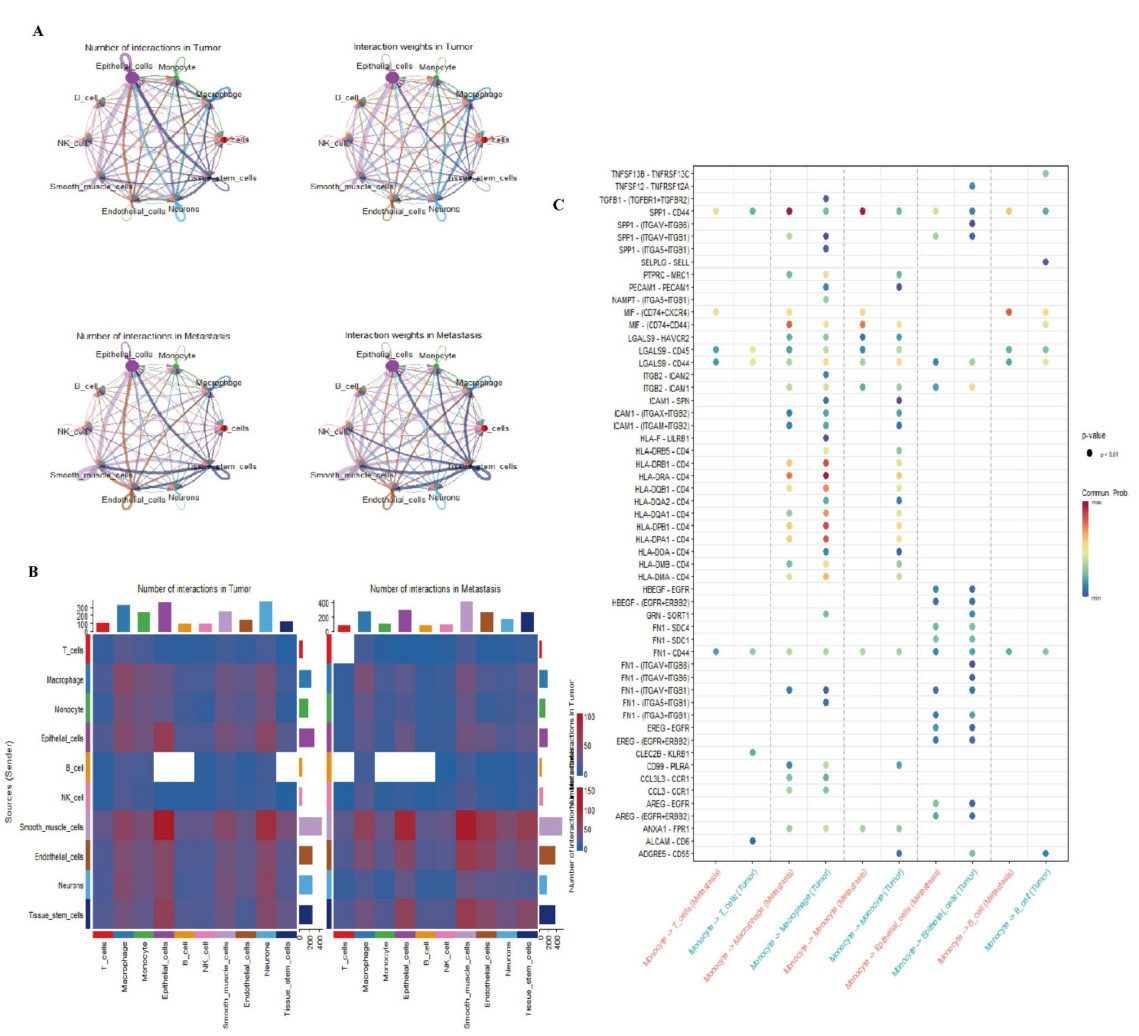
A illustrates the communication networks among different cell subpopulations in tumor and metastatic tissues, highlighting both the quantity and weight of these communications. The upper left and upper right panels depict the communication quantities and weights within tumor tissues, while the lower left and lower right panels present the same analyses for metastatic tissues. Different colored lines represent the strength of communication between cell subpopulations, with thicker lines indicating more frequent interactions.Figure B presents a heatmap showcasing the communication quantities between various cell subpopulations in tumor and metastatic tissues, with tumor tissue on the left and metastatic tissue on the right.Figure C displays the interaction patterns of key ligand-receptor pairs in tumor and metastatic tissues. The color intensity indicates the significance of these interactions, while the size of the circles represents the probability of communication

In this study, we utilized CellChat to analyze intercellular communication networks in tumor and metastatic tissues. By categorizing cells into different subpopulations (such as epithelial cells, monocytes, and macrophages), we inferred the quantity and weight of communication among these subpopulations. The results revealed significant differences in the interaction network structures between tumor and metastatic tissues. In tumor tissues, epithelial cells communicated most frequently and with higher weight with immune cells (such as T cells and macrophages) (Fig. 14A), suggesting a crucial role for epithelial cells in modulating the tumor immune microenvironment.In contrast, the quantity of communication between epithelial and immune cells decreased in metastatic tissues, although the weight of certain key interactions significantly increased, indicating that specific signaling pathways may be reinforced to enhance the invasiveness and metastatic capability of tumors during the metastatic process.Heatmap analysis (Fig. 14B) further illustrated the differences in communication quantities among various cell subpopulations. Notably, certain cell types (such as macrophages and monocytes) exhibited significant changes in their communication patterns and quantities in different environments, which may be associated with immune evasion and remodeling of the tumor microenvironment during metastasis.By analyzing the ligand-receptor pairs involved in intercellular communication within tumor and metastatic tissues, we identified several key pathways with significant functional differences (Fig. 14C). These ligand-receptor pairs displayed distinct interaction patterns across different tumor microenvironments, revealing their potential roles in tumor progression and metastasis.For instance, the TNF family signaling pathway—specifically, TNFSF10-TNFRSF10C and TNFSF15-TNFRSF12A—was significantly upregulated in metastatic tissues, suggesting a critical role for the TNF signaling pathway in promoting tumor cell invasion and anti-apoptosis. Activation of the TNF pathway is typically associated with inflammatory responses and cell survival, which may provide favorable conditions for tumor cells during metastasis.In the cell adhesion molecule signaling pathway, ICAM1-ITGAL and PECAM1-PECAM1 exhibited significant activity in metastatic tissues, indicating that cell adhesion molecules may facilitate tumor cell adhesion and infiltration, assisting their colonization in new tissues.

In the interleukin signaling pathway, IL6-IL6R and IL1B-IL1R1 were significantly upregulated in metastatic tissues. IL-6 and IL-1 pathways play vital roles in inflammatory responses and immune evasion, potentially promoting metastatic growth by modulating the tumor microenvironment.The chemokine signaling pathway CXCL12-CXCR4 was notably enhanced in metastatic tumors, typically associated with tumor cell migration and invasion, highlighting its importance in guiding tumor cells to new colonization sites.Lastly, growth factor signaling pathways, including EGF-EGFR and HGF-MET, were expressed in both tumor and metastatic tissues, but exhibited greater interaction intensity in metastatic tissues. These growth factor signaling pathways are closely linked to tumor growth and angiogenesis, potentially supporting tumor survival and expansion in distant organs by providing essential growth signals.

#### Clinical relevance analysis of SLC2A1

**Fig. 15:**
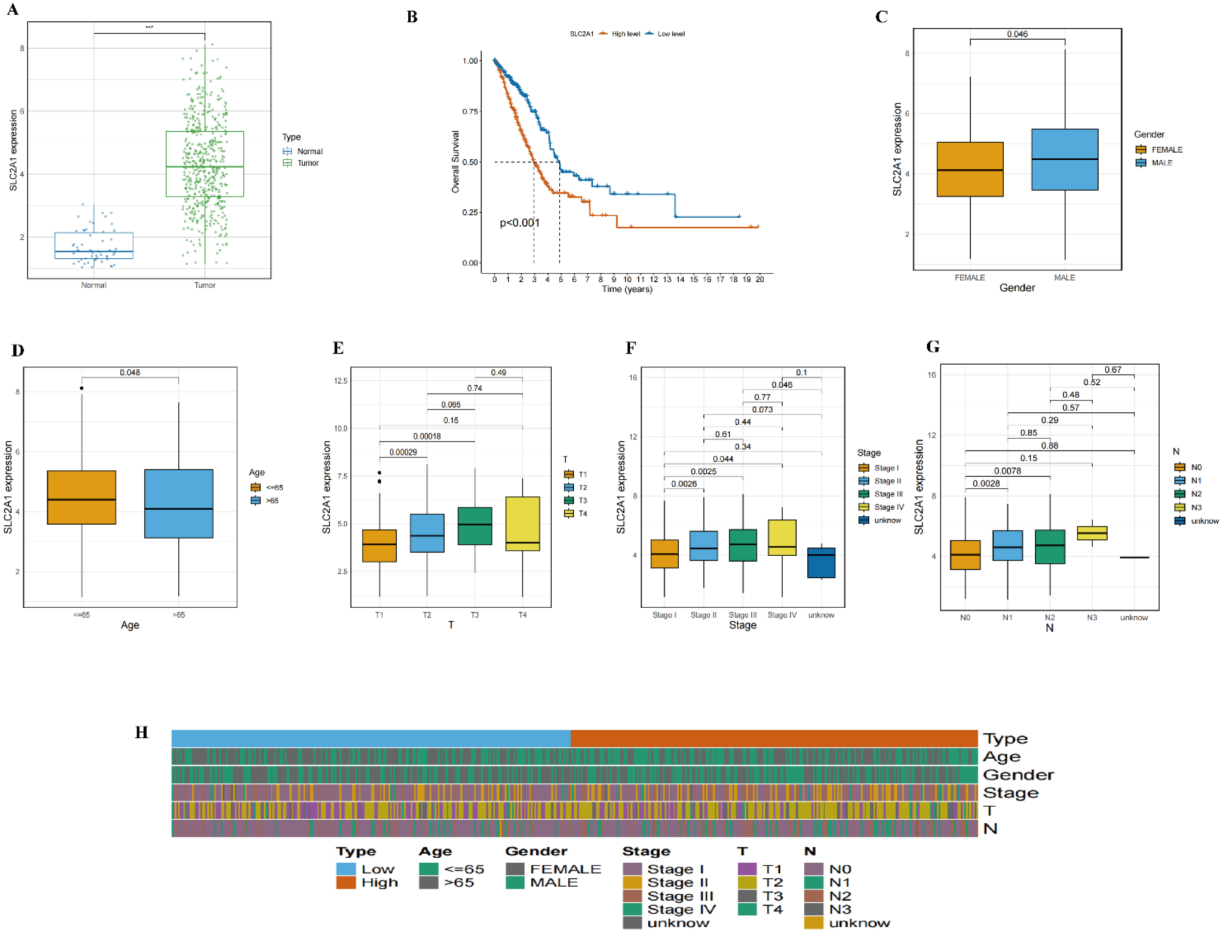
**A** illustrates the expression differences of SLC2A1 between normal and tumor tissues. Figure** B** demonstrates the relationship between high and low SLC2A1 expression and overall patient survival rates. Figures** C**-**G** show SLC2A1 expression levels across various factors, including gender, age, tumor size (T stage), tumor stage, and lymph node metastasis status (N stage). Figure** H** presents a heatmap correlating SLC2A1 expression with multiple clinical characteristics

Figure 15 A demonstrates a significant difference in SLC2A1 expression between normal and tumor tissues, with levels markedly higher in tumor tissues. This finding suggests a critical role for SLC2A1 in tumorigenesis and progression, supported by statistical analysis that confirms the significance of this expression difference (*p* < 0.001), indicating its potential as a tumor marker.In survival analysis, Fig. 15B reveals that patients with high SLC2A1 expression have significantly lower overall survival rates compared to those with low expression (*p* < 0.001), suggesting that elevated SLC2A1 levels may correlate with poor prognosis and serve as an important factor influencing patient survival.Gender analysis shows that Fig. 15C indicates male patients exhibit slightly higher SLC2A1 expression than female patients, with this difference being statistically significant (*p* = 0.046), suggesting that gender may play a role in regulating SLC2A1 expression. Figure 15D presents age analysis, indicating that patients over 65 years exhibit significantly higher SLC2A1 expression compared to younger patients (*p* = 0.048), potentially reflecting age-related biological changes in tumors.Regarding tumor size (T stage), Fig. 15 E illustrates a significant increase in SLC2A1 expression with tumor size, especially in T3 and T4 stages (*p* = 0.00018 and *p* = 0.00029), suggesting a close association with tumor invasiveness.In terms of tumor staging, Fig. 15F shows significantly elevated SLC2A1 expression in advanced stages (Stage III and Stage IV) (*p* = 0.0025 and *p* = 0.044), further supporting its potential as a marker for tumor progression.For lymph node status (N stage), Fig. 15G indicates that SLC2A1 expression is significantly higher in patients with lymph node metastasis (N1 and N2) compared to those without (N0) (*p* = 0.0028 and *p* = 0.0078), suggesting its important role in tumor metastasis.Comprehensive heatmap analysis reveals the relationship between SLC2A1 expression and various clinical features (such as age, gender, tumor stage, T stage, and N stage) (Fig. 15H). Through stratified analysis, we gain clearer insights into how these features influence SLC2A1 expression.

### Silencing SLC2A1 inhibits tumor cell proliferation and migration

**Fig. 16:**
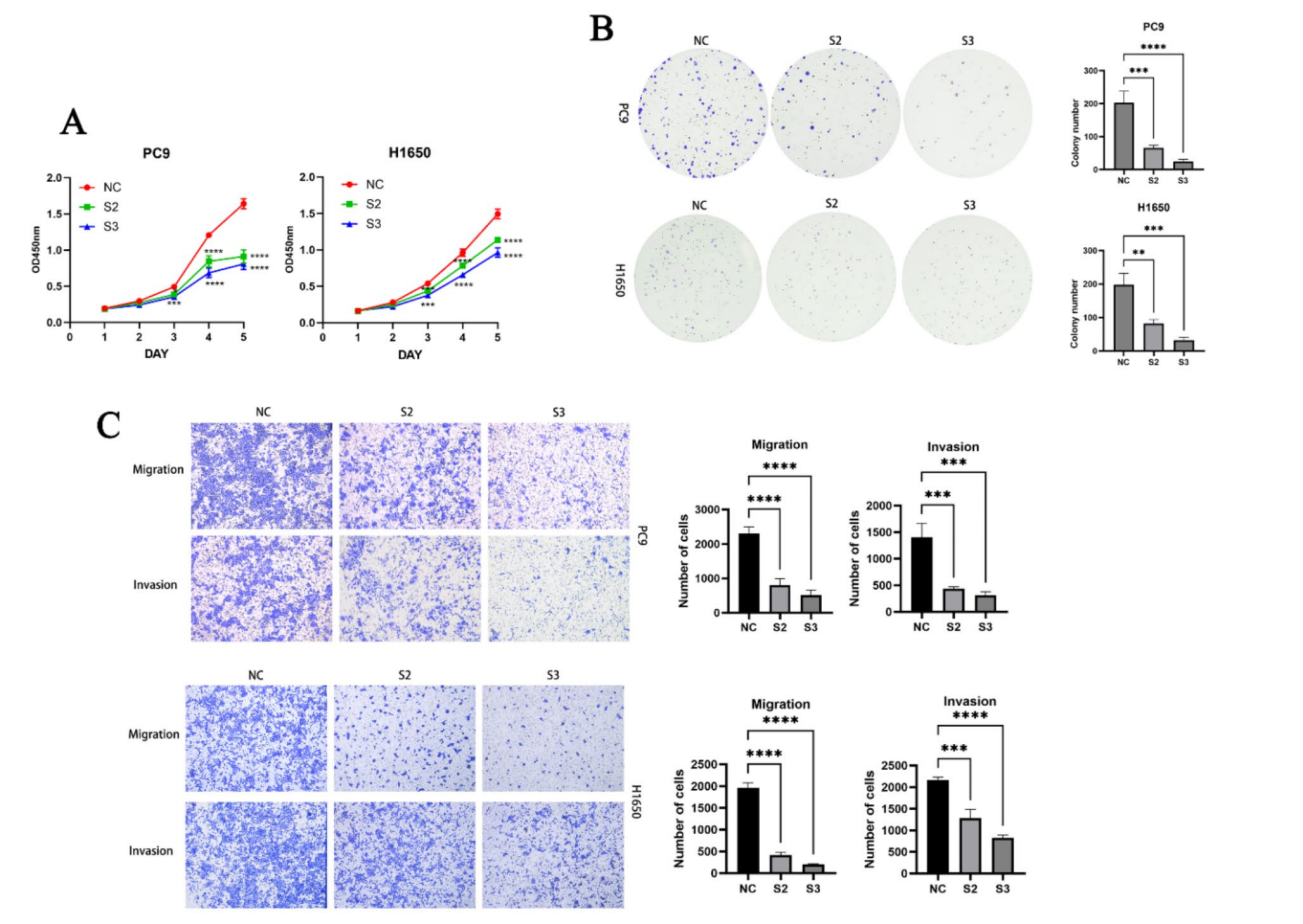
Knockdown of SLC2A1 inhibits LUAD cell proliferation, colony formation, migration, and invasion (**A**) CCK-8 assay results showing significantly reduced proliferation of PC9 and H1650 cells in the si-SLC2A1 groups (S2 and S3) compared to the negative control (NC). (**B**) Colony formation assay results indicating a significant decrease in the number of colonies formed by si-SLC2A1 cells compared to NC. Representative images and quantification are shown. (**C**) Transwell migration and invasion assays demonstrating reduced migratory and invasive capacities of si-SLC2A1 cells compared to NC. Representative images and quantification are presented. Statistical significance: *p* < 0.01 (), *p* < 0.001 (), *p* < 0.0001 (***).

To investigate the biological function of SLC2A1 in LUAD, we selected the lung adenocarcinoma cell lines PC9 and H1650, which have high SLC2A1 expression, and knocked down SLC2A1 expression in tumor cells using siRNA. A series of functional analyses were performed on the transfected cells. The CCK-8 assay results showed that the proliferation capacity of the si-SLC2A1 group (S2 and S3) was significantly reduced compared to the si-NC group (Fig. 16A). This result was further confirmed by the colony formation assay, where the number of colonies in the si-SLC2A1 group was significantly lower than in the control group (Fig. 16B). Furthermore, in the Transwell migration and invasion assays, the si-SLC2A1 group showed a significant reduction in the number of migrating and invading cells compared to the si-NC group (Fig. 16C). These results indicate that SLC2A1 functions as an oncogene, promoting LUAD cell proliferation, migration, and invasion.

**Silencing SLC2A1 activates the P53-P21 signaling pathway**.

**Fig. 17:**
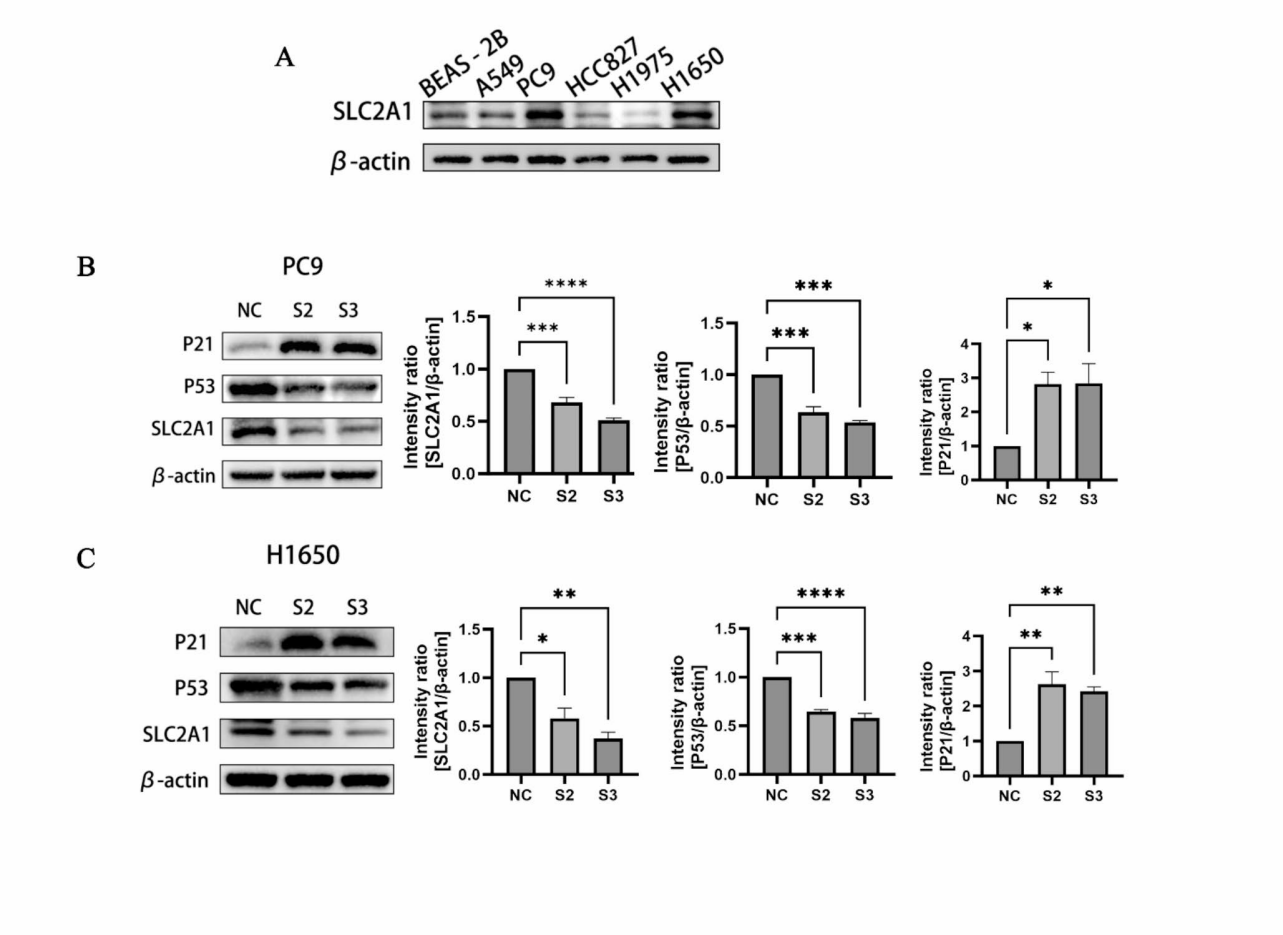
Silencing SLC2A1 activates the P53-P21 signaling pathway in LUAD cells. (**A**) Western blot analysis showing the expression of SLC2A1 in NSCLC cell lines (PC9, H1650, A549, HCC827, H1975) and the normal bronchial epithelial cell line BEAS-2B. (**B**, **C**) Western blot results for PC9 (**B**) and H1650 (**C**) cells after silencing SLC2A1 (S2 and S3), showing decreased P53 expression and significantly increased P21 expression compared to the negative control (NC). Quantifications of protein expression relative to β-actin are presented. Statistical significance: *p* < 0.05 (), *p* < 0.01 (), *p* < 0.001 (), *p* < 0.0001 (****)

To investigate the biological function of SLC2A1 in lung adenocarcinoma, we first evaluated SLC2A1 protein expression in multiple LUAD cell lines using Western blot analysis (Fig. 17A). The results showed that SLC2A1 was highly expressed in PC9 and H1650 cells, which were selected for subsequent experiments.Since silencing SLC2A1 inhibited the proliferation and migration of LUAD cells, we further performed Western blot analysis to identify signaling pathways and key molecules associated with cell proliferation, migration, and invasion. The results showed that, compared to the si-NC group, P53 expression was reduced, while P21 expression was significantly increased in SLC2A1-knockdown PC9 and H1650 lung adenocarcinoma cells (Fig. 17B, C). These findings suggest that silencing SLC2A1 may activate the P53-P21 signaling pathway, wherein P53 promotes P21 expression, and overexpression of P21 subsequently provides negative feedback to suppress P53 expression.Collectively, these results indicate that silencing SLC2A1 activates the P53-P21 signaling pathway, thereby inhibiting cell proliferation, migration, and invasion.

## Discussion

This study integrates single-cell RNA sequencing (scRNA-seq) with extensive genome-wide association study (GWAS) data to reveal the complex genetic characteristics of epithelial cells in lung adenocarcinoma and their crucial role in the tumor microenvironment. Our research offers a novel perspective for understanding the molecular mechanisms underlying lung adenocarcinoma and provides strong support for personalized therapeutic strategies.

Through scRNA-seq analysis, we have elucidated the intricate heterogeneity of epithelial cells in lung adenocarcinoma. These cells exhibit significant diversity, involving multiple key biological processes and signaling pathways, particularly in regulating cellular metabolism, proliferation, and immune evasion. We specifically focused on the expression levels of genes such as SLC2A1, F12, GOLM1, and PERP in epithelial cells. These genes play vital roles in lung adenocarcinoma, with their high expression closely linked to increased aggressiveness of cancer cells and poor prognosis. This finding not only aligns with our research results but also corroborates observations from other cancer studies. Notably, the elevated expression of SLC2A1, which encodes glucose transporter 1 (GLUT1), enhances cancer cell uptake of glucose, thereby reprogramming cellular metabolic pathways (Li et al. 2023). Through this metabolic reprogramming, cancer cells can survive in nutrient-depleted environments and maintain rapid proliferation (Zheng et al. 2022). Our study further reveals that epithelial cells with high SLC2A1 expression are more prevalent at the tumor margins and metastatic sites, suggesting their potential critical role in tumor dissemination and metastasis. This indicates that SLC2A1 is not only a regulator of tumor cell metabolism but may also directly promote cancer progression by participating in cellular migration and invasion.

Additionally, our research uncovers the complex interaction network between immune cells and epithelial cells within the tumor microenvironment. Through Bayesian deconvolution analysis, we identified certain genetic variations that may influence the functionality of immune cells, thereby altering their response to tumor cells. These interaction networks encompass not only the direct responses of immune cells to tumor cells but also the regulation of immune evasion mechanisms. These findings provide novel insights into the immune evasion mechanisms of cancer and lay a theoretical foundation for the development of innovative immunotherapeutic strategies. We observed rich signaling pathways between immune cells, such as T cells and macrophages, and epithelial cells in the tumor microenvironment. These interactions may affect immune cell infiltration and functionality by regulating the expression of cytokines and chemokines. Specifically, we noted a significant upregulation of key immune checkpoint molecules such as PD-L1 and CTLA-4 in samples with high epithelial cell expression, indicating that these tumors may exhibit a robust immunosuppressive microenvironment, thereby hindering effective immune responses. This finding holds important clinical implications for the formulation and optimization of immunotherapy strategies targeting lung adenocarcinoma.

To further explore the association between genes and clinical prognosis, we employed various machine learning algorithms to construct efficient prognostic prediction models. These models can identify core genes closely associated with lung adenocarcinoma prognosis, such as F12, GOLM1, and S100P. The gene F12, which encodes coagulation factor XII (Hageman factor), plays a crucial role in initiating the intrinsic coagulation pathway. This gene is involved not only in coagulation processes but also in the bradykinin-kinin system, influencing vasodilation, inflammatory responses, and blood pressure regulation (Biswas et al. 2016). In cancer research, high expression of F12 is associated with tumor progression and poor prognosis (Cheishvili et al. 2023). Studies suggest that F12 may participate in cancer dissemination and metastasis by regulating tumor angiogenesis and promoting the migration of tumor cells. The GOLM1 gene encodes Golgi membrane protein 1 (GP73), a membrane protein localized in the Golgi apparatus involved in protein modification, transport, and secretion (Sui et al. 2021). GOLM1 is expressed at low levels in normal cells but is significantly upregulated in various cancers (e.g., liver, lung, prostate), with its expression level closely linked to tumor aggressiveness and poor prognosis (Sui et al. 2021; Song et al. 2021; Yamoah et al. 2015). The S100P gene belongs to the S100 protein family, encoding a calcium-binding protein. Members of the S100 protein family play important roles in cell proliferation, differentiation, apoptosis, energy metabolism, inflammation, and cell cycle regulation (Camara et al. 2020). S100P is highly expressed in multiple tumor types, particularly in pancreatic, breast, colorectal, and lung adenocarcinomas, where its expression correlates with cancer invasiveness and metastasis (Cong et al. 2020; Ahmed et al. 2023; Hsu et al. 2015). F12 (Factor XII), a key factor in the blood coagulation process, has been found to be closely related to the coagulation system, angiogenesis, and inflammatory response within the tumor microenvironment (Pan et al. 2023). In lung adenocarcinoma, the upregulation of F12 may promote tumor angiogenesis and metastasis by activating endogenous coagulation pathways (Crespo-Bravo et al. 2024). Furthermore, F12 may be closely associated with immune cell recruitment, particularly in the context of tumor immune evasion mechanisms, as it affects immune cell function and tumor microenvironment immune escape via coagulation pathways (Campello et al. 2018).GOLM1 (Golgi membrane protein 1) is a protein associated with tumor cell metabolism, cell cycle regulation, and cell migration (Chen et al. 2021). In lung adenocarcinoma, high expression of GOLM1 is generally correlated with increased tumor cell proliferation and invasiveness (By regulating the expression of matrix metalloproteinases (MMPs), GOLM1 facilitates the degradation of the extracellular matrix (ECM), enabling tumor cells to breach the matrix barrier and invade surrounding tissues. Additionally, GOLM1 may further promote tumor invasion and metastasis by affecting the expression of ECM components and cell adhesion molecules within the tumor microenvironment (Sun et al. 2024).S100P (S100 calcium-binding protein P) is a typical calcium-binding protein that has been shown to play a crucial role in various tumor types, particularly in tumor cell migration, invasion, and immune evasion (Xu et al. 2024). In lung adenocarcinoma, upregulation of S100P is closely associated with cytoskeletal reorganization, tumor metastasis, and immune evasion (Fan et al. 2024). Specifically, it regulates immune cell functions, such as T-cell suppression, macrophage polarization, and activation of immune checkpoints, thereby promoting tumor immune evasion (Gao et al. 2024).

These genes have demonstrated high stability and predictive power across various prognostic models. The application of machine learning techniques has significantly enhanced our ability to process complex biological data and provides a powerful tool for discovering new biomarkers. Through cross-validation and validation across multiple datasets, our models performed exceptionally well in diverse populations, showcasing their accuracy and robustness, indicating promising prospects for clinical application. A major innovation of this study lies in the successful integration of scRNA-seq and GWAS data, providing a more comprehensive perspective for elucidating the molecular mechanisms of lung adenocarcinoma. By applying Bayesian deconvolution technology, we accurately dissected cell-type-specific gene expression within mixed tissue samples, offering strong support for understanding intercellular interactions and further advancing research on the interactions between different cell populations within the tumor microenvironment. This integrative approach opens new avenues for lung adenocarcinoma research and provides important references for future investigations. Our experiments also demonstrate that SLC2A1 functions as an oncogene by promoting tumor cell proliferation, migration, and invasion. Silencing SLC2A1 activates the P53-P21 signaling pathway, thereby inhibiting cell growth and metastasis.

However, the study also has certain limitations. First, the sample size is limited, which may affect the generalizability and applicability of the results. Second, although we have identified some potential therapeutic targets and biomarkers, the clinical applicability of these findings requires further validation. Future research should expand the sample size and incorporate clinical data for more comprehensive validation to enhance the applicability of the results. Nevertheless, the application of machine learning in biomedical research still faces challenges, such as data quality and model interpretability issues. Future studies should focus on improving data diversity and quality while incorporating biological experimental validation to enhance the practicality and interpretability of the models.

Based on the findings of this study, future research can proceed in several directions: Sample Diversification and Expansion: Increasing the sample size and including data from different populations and subtypes of lung adenocarcinoma will help uncover more genetic characteristics and molecular mechanisms.Multi-Omics Data Integration: In addition to scRNA-seq and GWAS data, integrating proteomics, metabolomics, and other data could provide a more comprehensive biological perspective.Clinical Application Validation: Applying laboratory findings to clinical trials to validate the efficacy and safety of newly identified biomarkers and therapeutic targets.Development of Novel Therapeutic Strategies: Based on identified key genes and signaling pathways, developing new targeted drugs and immunotherapy strategies to improve treatment outcomes for lung adenocarcinoma.

In conclusion, this study, through multi-omics integration analysis, provides new insights into the genetic and molecular characteristics of lung adenocarcinoma. These findings not only deepen our understanding of the disease but also offer directions for future research and therapeutic strategies. We anticipate that future studies will further expand on these results to improve the survival rates and quality of life for lung adenocarcinoma patients.

## Supplementary Information

Supplementary Information 1.

## Data Availability

This study analyzed publicly available data sets. All original analytical datasets used or analyzed during the current study are available from the corresponding author on reasonable request.

## References

[CR40] Ahmed AA, Greenhalf W, Palmer DH, Williams N, Worthington J, Arshad T, Haider S, Alexandrou E, Guneri D, Waller ZAE, Neidle S (2023) The potent G-Quadruplex-binding compound QN-302 downregulates S100P gene expression in cells and in an in vivo model of pancreatic cancer. Molecules 28:2452. 10.3390/molecules28062452 36985425 10.3390/molecules28062452 PMC10051992

[CR33] Biswas N, Maihofer AX, Mir SA, Rao F, Zhang K, Khandrika S, Mahata M, Friese RS, Hightower CM, Mahata SK, Baker DG, Nievergelt CM, Vaingankar SM, O’Connor DT (2016) Polymorphisms at the F12 and KLKB1 loci have significant trait association with activation of the renin-angiotensin system. BMC Med Genet 17:21. 10.1186/s12881-016-0283-5 26969407 10.1186/s12881-016-0283-5 PMC4788869

[CR23] Butler A, Hoffman P, Smibert P, Papalexi E, Satija R (2018) Integrating single-cell transcriptomic data across different conditions, technologies, and species. Nat Biotechnol 36:411–420. 10.1038/nbt.4096 29608179 10.1038/nbt.4096 PMC6700744

[CR38] Camara R, Ogbeni D, Gerstmann L, Ostovar M, Hurer E, Scott M, Mahmoud NG, Radon T, Crnogorac-Jurcevic T, Patel P, Mackenzie LS, Chau DYS, Kirton SB, Rossiter S (2020) Discovery of novel small molecule inhibitors of S100P with in vitro anti-metastatic effects on pancreatic cancer cells. Eur J Med Chem 203:112621. 10.1016/j.ejmech.2020.112621 32707527 10.1016/j.ejmech.2020.112621 PMC7501730

[CR44] Campello E, Henderson MW, Noubouossie DF, Simioni P, Key NS (2018) Contact system activation and Cancer: new insights in the pathophysiology of cancer-associated thrombosis. Thromb Haemost 118:251–265. 10.1160/TH17-08-0596 29378353 10.1160/TH17-08-0596

[CR34] Cheishvili D, Wong C, Karim MM, Kibria MG, Jahan N, Das PC, Yousuf MAK, Islam MA, Das DC, Noor-E-Alam SM, Szyf M, Alam S, Khan WA, Al Mahtab M (2023) A high-throughput test enables specific detection of hepatocellular carcinoma. Nat Commun 14:3306. 10.1038/s41467-023-39055-7 37286539 10.1038/s41467-023-39055-7 PMC10247794

[CR45] Chen J, Lin Z, Liu L, Zhang R, Geng Y, Fan M, Zhu W, Lu M, Lu L, Jia H, Zhang J, Qin L-X (2021) GOLM1 exacerbates CD8+ T cell suppression in hepatocellular carcinoma by promoting exosomal PD-L1 transport into tumor-associated macrophages. Signal Transduct Target Ther 6:397. 10.1038/s41392-021-00784-0 34795203 10.1038/s41392-021-00784-0 PMC8602261

[CR28] Chu T, Wang Z, Pe’er D, Danko CG (2022) Cell type and gene expression deconvolution with BayesPrism enables bayesian integrative analysis across bulk and single-cell RNA sequencing in oncology. Nat Cancer 3:505–517. 10.1038/s43018-022-00356-3 35469013 10.1038/s43018-022-00356-3 PMC9046084

[CR26] Colaprico A, Silva TC, Olsen C, Garofano L, Cava C, Garolini D, Sabedot TS, Malta TM, Pagnotta SM, Castiglioni I, Ceccarelli M, Bontempi G, Noushmehr H (2016) TCGAbiolinks: an R/Bioconductor package for integrative analysis of TCGA data. Nucleic Acids Res 44:e71. 10.1093/nar/gkv1507 26704973 10.1093/nar/gkv1507 PMC4856967

[CR39] Cong Y, Cui Y, Wang S, Jiang L, Cao J, Zhu S, Birkin E, Lane J, Ruge F, Jiang WG, Qiao G (2020) Calcium-binding protein S100P promotes tumor progression but enhances chemosensitivity in breast cancer. Front Oncol 10:566302. 10.3389/fonc.2020.566302 33042844 10.3389/fonc.2020.566302 PMC7522638

[CR13] Cords L, Engler S, Haberecker M, Rüschoff JH, Moch H, de Souza N, Bodenmiller B (2024) Cancer-associated fibroblast phenotypes are associated with patient outcome in non-small cell lung cancer. Cancer Cell 42:396–412e5. 10.1016/j.ccell.2023.12.021 38242124 10.1016/j.ccell.2023.12.021 PMC10929690

[CR43] Crespo-Bravo M, Hettich A, Thorlacius-Ussing J, Cox TR, Karsdal MA, Willumsen N (2024) Type XII collagen is elevated in serum from patients with solid tumors: a non-invasive biomarker of activated fibroblasts. Clin Exp Med 24:166. 10.1007/s10238-024-01431-y 39048763 10.1007/s10238-024-01431-y PMC11269340

[CR24] Cui G, Li S, Ye H, Yang Y, Jia X, Lin M, Chu Y, Feng Y, Wang Z, Shi Z, Zhang X (2023) Gut microbiome and frailty: insight from genetic correlation and mendelian randomization. Gut Microbes 15:2282795. 10.1080/19490976.2023.2282795 37990415 10.1080/19490976.2023.2282795 PMC10730212

[CR7] Dai J, Lv J, Zhu M, Wang Y, Qin N, Ma H, He Y-Q, Zhang R, Tan W, Fan J, Wang T, Zheng H, Sun Q, Wang L, Huang M, Ge Z, Yu C, Guo Y, Wang T-M, Wang J, Xu L, Wu W, Chen L, Bian Z, Walters R, Millwood IY, Li X-Z, Wang X, Hung RJ, Christiani DC, Chen H, Wang M, Wang C, Jiang Y, Chen K, Chen Z, Jin G, Wu T, Lin D, Hu Z, Amos CI, Wu C, Wei Q, Jia W-H, Li L, Shen H (2019) Identification of risk loci and a polygenic risk score for lung cancer: a large-scale prospective cohort study in Chinese populations. Lancet Respir Med 7:881–891. 10.1016/S2213-2600(19)30144-4 31326317 10.1016/S2213-2600(19)30144-4 PMC7015703

[CR22] Dhainaut M, Rose SA, Akturk G, Wroblewska A, Nielsen SR, Park ES, Buckup M, Roudko V, Pia L, Sweeney R, Le Berichel J, Wilk CM, Bektesevic A, Lee BH, Bhardwaj N, Rahman AH, Baccarini A, Gnjatic S, Pe’er D, Merad M, Brown BD (2022) Spatial CRISPR genomics identifies regulators of the tumor microenvironment. Cell 185:1223–1239e20. 10.1016/j.cell.2022.02.015 35290801 10.1016/j.cell.2022.02.015 PMC8992964

[CR49] Fan G, Xie T, Yang M, Li L, Tang L, Han X, Shi Y (2024) Spatial analyses revealed S100P+ TFF1+ tumor cells in spread through air spaces samples correlated with undesirable therapy response in non-small cell lung cancer. J Transl Med 22:917. 10.1186/s12967-024-05722-6 39385235 10.1186/s12967-024-05722-6 PMC11462816

[CR10] Feng X (2023) Integrative analysis of GWAS and transcriptomics data reveal key genes for non-small lung cancer. Med Oncol 40:270. 10.1007/s12032-023-02139-x 37592093 10.1007/s12032-023-02139-x

[CR50] Gao L, Bai Y, Zhou J, Liang C, Dong Y, Han T, Liu Y, Guo J, Wu J, Hu D (2024) S100P facilitates LUAD progression via PKA/c-Jun-mediated tumor-associated macrophage recruitment and polarization. Cell Signal 120:111179. 10.1016/j.cellsig.2024.111179 38640980 10.1016/j.cellsig.2024.111179

[CR4] Hirz T, Mei S, Sarkar H, Kfoury Y, Wu S, Verhoeven BM, Subtelny AO, Zlatev DV, Wszolek MW, Salari K, Murray E, Chen F, Macosko EZ, Wu C-L, Scadden DT, Dahl DM, Baryawno N, Saylor PJ, Kharchenko PV, Sykes DB (2023) Dissecting the immune suppressive human prostate tumor microenvironment via integrated single-cell and spatial transcriptomic analyses. Nat Commun 14:663. 10.1038/s41467-023-36325-2 36750562 10.1038/s41467-023-36325-2 PMC9905093

[CR41] Hsu Y-L, Hung J-Y, Liang Y-Y, Lin Y-S, Tsai M-J, Chou S-H, Lu C-Y, Kuo P-L (2015) S100P interacts with integrin α7 and increases cancer cell migration and invasion in lung cancer. Oncotarget 6:29585–29598. 10.18632/oncotarget.4987 26320193 10.18632/oncotarget.4987 PMC4745748

[CR8] Kim N, Kim HK, Lee K, Hong Y, Cho JH, Choi JW, Lee J-I, Suh Y-L, Ku BM, Eum HH, Choi S, Choi Y-L, Joung J-G, Park W-Y, Jung HA, Sun J-M, Lee S-H, Ahn JS, Park K, Ahn M-J, Lee H-O (2020) Single-cell RNA sequencing demonstrates the molecular and cellular reprogramming of metastatic lung adenocarcinoma. Nat Commun 11:2285. 10.1038/s41467-020-16164-1 32385277 10.1038/s41467-020-16164-1 PMC7210975

[CR19] Li Q, Wang R, Yang Z, Li W, Yang J, Wang Z, Bai H, Cui Y, Tian Y, Wu Z, Guo Y, Xu J, Wen L, He J, Tang F, Wang J (2022) Molecular profiling of human non-small cell lung cancer by single-cell RNA-seq. Genome Med 14:87. 10.1186/s13073-022-01089-9 35962452 10.1186/s13073-022-01089-9 PMC9375433

[CR31] Li Y, Tang S, Shi X, Lv J, Wu X, Zhang Y, Wang H, He J, Zhu Y, Ju Y, Zhang Y, Guo S, Yang W, Yin H, Chen L, Gao D, Jin G (2023) Metabolic classification suggests the GLUT1/ALDOB/G6PD axis as a therapeutic target in chemotherapy-resistant pancreatic cancer. Cell Rep Med 4:101162. 10.1016/j.xcrm.2023.101162 37597521 10.1016/j.xcrm.2023.101162 PMC10518604

[CR30] Liu H, Zhang W, Zhang Y, Adegboro AA, Fasoranti DO, Dai L, Pan Z, Liu H, Xiong Y, Li W, Peng K, Wanggou S, Li X (2024) Mime: a flexible machine-learning framework to construct and visualize models for clinical characteristics prediction and feature selection, Comput Struct Biotechnol J 23 2798–2810. 10.1016/j.csbj.2024.06.035 10.1016/j.csbj.2024.06.035 PMC11269309 39055398

[CR27] Love MI, Huber W, Anders S (2014) Moderated estimation of Fold change and dispersion for RNA-seq data with DESeq2. Genome Biol 15:550. 10.1186/s13059-014-0550-8 25516281 10.1186/s13059-014-0550-8 PMC4302049

[CR25] Ma Y, Deng C, Zhou Y, Zhang Y, Qiu F, Jiang D, Zheng G, Li J, Shuai J, Zhang Y, Yang J, Su J (2023) Polygenic regression uncovers trait-relevant cellular contexts through pathway activation transformation of single-cell RNA sequencing data. Cell Genomics 3:100383. 10.1016/j.xgen.2023.100383 37719150 10.1016/j.xgen.2023.100383 PMC10504677

[CR29] Maeser D, Gruener RF, Huang RS (2021) oncoPredict: an R package for predicting in vivo or cancer patient drug response and biomarkers from cell line screening data. Brief Bioinform 22:bbab260. 10.1093/bib/bbab260 34260682 10.1093/bib/bbab260 PMC8574972

[CR5] Mao S, Wang Y, Chao N, Zeng L, Zhang L (2024) Integrated analysis of single-cell RNA-seq and bulk RNA-seq reveals immune suppression subtypes and establishes a novel signature for determining the prognosis in lung adenocarcinoma. Cell Oncol (Dordr). 10.1007/s13402-024-00948-4 38616208 10.1007/s13402-024-00948-4 PMC12974075

[CR15] Marjanovic ND, Hofree M, Chan JE, Canner D, Wu K, Trakala M, Hartmann GG, Smith OC, Kim JY, Evans KV, Hudson A, Ashenberg O, Porter CBM, Bejnood A, Subramanian A, Pitter K, Yan Y, Delorey T, Phillips DR, Shah N, Chaudhary O, Tsankov A, Hollmann T, Rekhtman N, Massion PP, Poirier JT, Mazutis L, Li R, Lee J-H, Amon A, Rudin CM, Jacks T, Regev A, Tammela T (2020) Emergence of a high-plasticity cell state during Lung Cancer Evolution. Cancer Cell 38:229–246e13. 10.1016/j.ccell.2020.06.012 32707077 10.1016/j.ccell.2020.06.012 PMC7745838

[CR3] McKay JD, Hung RJ, Han Y, Zong X, Carreras-Torres R, Christiani DC, Caporaso NE, Johansson M, Xiao X, Li Y, Byun J, Dunning A, Pooley KA, Qian DC, Ji X, Liu G, Timofeeva MN, Bojesen SE, Wu X, Le Marchand L, Albanes D, Bickeböller H, Aldrich MC, Bush WS, Tardon A, Rennert G, Teare MD, Field JK, Kiemeney LA, Lazarus P, Haugen A, Lam S, Schabath MB, Andrew AS, Shen H, Hong Y-C, Yuan J-M, Bertazzi PA, Pesatori AC, Ye Y, Diao N, Su L, Zhang R, Brhane Y, Leighl N, Johansen JS, Mellemgaard A, Saliba W, Haiman CA, Wilkens LR, Fernandez-Somoano A, Fernandez-Tardon G, van der Heijden HFM, Kim JH, Dai J, Hu Z, Davies MP, Marcus MW, Brunnström H, Manjer J, Melander O, Muller DC, Overvad K, Trichopoulou A, Tumino R, Doherty JA, Barnett MP, Chen C, Goodman GE, Cox A, Taylor F, Woll P, Brüske I, Wichmann H-E, Manz J, Muley TR, Risch A, Rosenberger A, Grankvist K, Johansson M, Shepherd FA, Tsao M-S, Arnold SM, Haura EB, Bolca C, Holcatova I, Janout V, Kontic M, Lissowska J, Mukeria A, Ognjanovic S, Orlowski TM, Scelo G, Swiatkowska B, Zaridze D, Bakke P, Skaug V, Zienolddiny S, Duell EJ, Butler LM, Koh W-P, Gao Y-T, Houlston RS, McLaughlin J, Stevens VL, Joubert P, Lamontagne M, Nickle DC, Obeidat M, Timens W, Zhu B, Song L, Kachuri L, Artigas MS, Tobin MD, Wain LV, Rafnar T, Thorgeirsson TE, Reginsson GW, Stefansson K, Hancock DB, Bierut LJ, Spitz MR, Gaddis NC, Lutz SM, Gu F, Johnson EO, Kamal A, Pikielny C, Zhu D, Lindströem S, Jiang X, Tyndale RF, Chenevix-Trench G, Beesley J, Bossé Y, Chanock S (2017) P. Brennan, M.T. Landi, C.I. Amos, Large-scale association analysis identifies new lung cancer susceptibility loci and heterogeneity in genetic susceptibility across histological subtypes, Nat Genet 49: 1126–1132. 10.1038/ng.3892 10.1038/ng.3892 PMC5510465 28604730

[CR16] Montégut L, Liu P, Zhao L, Pérez-Lanzón M, Chen H, Mao M, Zhang S, Derosa L, Naour JL, Lambertucci F, Mingoia S, Nogueira-Recalde U, Mena-Osuna R, Herranz-Montoya I, Djouder N, Baulande S, Pan H, Joseph A, Messaoudene M, Routy B, Fidelle M, Ahmed TB, Caron O, Busson P, Boulate D, Deschasaux-Tanguy M, Arnault N, Pol JG, Piaggio E, Touvier M, Zitvogel L, Delaloge S, Martins I, Kroemer G (2024) Acyl-coenzyme a binding protein (ACBP)--a risk factor for cancer diagnosis and an inhibitor of immunosurveillance. Mol Cancer 23:187. 10.1186/s12943-024-02098-5 39242519 10.1186/s12943-024-02098-5 PMC11378439

[CR21] Palermo B, Franzese O, Frisullo G, D’Ambrosio L, Panetta M, Campo G, D’Andrea D, Sperduti I, De Nicola F, Goeman F, Gallina F, Visca P, Facciolo F, Nisticò P (2023) CD28/PD1 co-expression: dual impact on CD8+ T cells in peripheral blood and tumor tissue, and its significance in NSCLC patients’ survival and ICB response. J Exp Clin Cancer Res 42:287. 10.1186/s13046-023-02846-3 37898752 10.1186/s13046-023-02846-3 PMC10612243

[CR42] Pan X, Zhang W, Wang L, Guo H, Zheng M, Wu H, Weng Q, He Q, Ding L, Yang B (2023) KLF12 transcriptionally regulates PD-L1 expression in non-small cell lung cancer. Mol Oncol 17:2659–2674. 10.1002/1878-0261.13512 37606530 10.1002/1878-0261.13512 PMC10701771

[CR11] Passaro A, Brahmer J, Antonia S, Mok T, Peters S (2022) Managing resistance to Immune checkpoint inhibitors in Lung Cancer: treatment and novel strategies. JCO 40:598–610. 10.1200/JCO.21.01845 10.1200/JCO.21.01845 34985992

[CR14] Qiao M, Zhou F, Liu X, Jiang T, Wang H, Li X, Zhao C, Cheng L, Chen X, Ren S, Wang Z, Zhou C (2024) Targeting focal adhesion kinase boosts immune response in KRAS/LKB1 co-mutated lung adenocarcinoma via remodeling the tumor microenvironment. Exp Hematol Oncol 13:11. 10.1186/s40164-023-00471-6 38291516 10.1186/s40164-023-00471-6 PMC10826079

[CR9] Ren Y, Wu R, Li C, Liu L, Li L, Weng S, Xu H, Xing Z, Zhang Y, Wang L, Liu Z, Han X (2024) Single-cell RNA sequencing integrated with bulk RNA sequencing analysis identifies a tumor immune microenvironment-related lncRNA signature in lung adenocarcinoma. BMC Biol 22:69. 10.1186/s12915-024-01866-5 38519942 10.1186/s12915-024-01866-5 PMC10960411

[CR2] Shi J, Shiraishi K, Choi J, Matsuo K, Chen T-Y, Dai J, Hung RJ, Chen K, Shu X-O, Kim YT, Landi MT, Lin D, Zheng W, Yin Z, Zhou B, Song B, Wang J, Seow WJ, Song L, Chang I-S, Hu W, Chien L-H, Cai Q, Hong Y-C, Kim HN, Wu Y-L, Wong MP, Richardson BD, Funderburk KM, Li S, Zhang T, Breeze C, Wang Z, Blechter B, Bassig BA, Kim JH, Albanes D, Wong JYY, Shin M-H, Chung LP, Yang Y, An S-J, Zheng H, Yatabe Y, Zhang X-C, Kim Y-C, Caporaso NE, Chang J, Ho JCM, Kubo M, Daigo Y, Song M, Momozawa Y, Kamatani Y, Kobayashi M, Okubo K, Honda T, Hosgood DH, Kunitoh H, Patel H, Watanabe S, Miyagi Y, Nakayama H, Matsumoto S, Horinouchi H, Tsuboi M, Hamamoto R, Goto K, Ohe Y, Takahashi A, Goto A, Minamiya Y, Hara M, Nishida Y, Takeuchi K, Wakai K, Matsuda K, Murakami Y, Shimizu K, Suzuki H, Saito M, Ohtaki Y, Tanaka K, Wu T, Wei F, Dai H, Machiela MJ, Su J, Kim YH, Oh I-J, Lee VHF, Chang G-C, Tsai Y-H, Chen K-Y, Huang M-S, Su W-C, Chen Y-M, Seow A, Park JY, Kweon S-S, Chen K-C, Gao Y-T, Qian B, Wu C, Lu D, Liu J, Schwartz AG, Houlston R, Spitz MR, Gorlov IP, Wu X, Yang P, Lam S, Tardon A, Chen C, Bojesen SE, Johansson M, Risch A, Bickeböller H, Ji B-T, Wichmann H-E, Christiani DC, Rennert G, Arnold S, Brennan P, McKay J, Field JK, Shete SS, Le Marchand L, Liu G, Andrew A, Kiemeney LA, Zienolddiny-Narui S, Grankvist K, Johansson M, Cox A, Taylor F, Yuan J-M, Lazarus P, Schabath MB, Aldrich MC, Jeon H-S, Jiang SS, Sung JS, Chen C-H, Hsiao C-F, Jung YJ, Guo H, Hu Z, Burdett L, Yeager M, Hutchinson A, Hicks B, Liu J, Zhu B, Berndt SI, Wu W, Wang J, Li Y, Choi JE, Park KH, Sung SW, Liu L, Kang CH, Wang W-C, Xu J, Guan P, Tan W, Yu C-J, Yang G, Sihoe ADL, Chen Y, Choi YY, Kim JS, Yoon H-I, Park IK, Xu P, He Q, Wang C-L, Hung H-H, Vermeulen RCH, Cheng I, Wu J, Lim W-Y, Tsai F-Y, Chan JKC, Li J, Chen H, Lin H-C, Jin L, Liu J, Sawada N, Yamaji T, Wyatt K, Li SA, Ma H, Zhu M, Wang Z, Cheng S, Li X, Ren Y, Chao A, Iwasaki M, Zhu J, Jiang G, Fei K, Wu G, Chen C-Y, Chen C-J, Yang P-C, Yu J, Stevens VL, Fraumeni JF, Chatterjee N, Gorlova OY, Hsiung CA, Amos CI, Shen H, Chanock SJ, Rothman N, Kohno T, Lan Q (2023), Genome-wide association study of lung adenocarcinoma in East Asia and comparison with a European population. Nat Commun 14: 3043, 10.1038/s41467-023-38196-z 37236969 10.1038/s41467-023-38196-z PMC10220065

[CR12] Sinjab A, Han G, Treekitkarnmongkol W, Hara K, Brennan PM, Dang M, Hao D, Wang R, Dai E, Dejima H, Zhang J, Bogatenkova E, Sanchez-Espiridion B, Chang K, Little D, Bazzi S, Tran LM, Krysan K, Behrens C, Duose DY, Parra ER, Raso MG, Solis LM, Fukuoka J, Zhang J, Sepesi B, Cascone T, Byers LA, Gibbons DL, Chen J, Moghaddam SJ, Ostrin EJ, Rosen D, Heymach JV, Scheet P, Dubinett SM, Fujimoto J, Wistuba II, Stevenson CS, Spira A, Wang L, Kadara H (2021) Resolving the spatial and cellular architecture of lung adenocarcinoma by multi-region single-cell sequencing. Cancer Discov 11:2506–2523. 10.1158/2159-8290.CD-20-1285 33972311 10.1158/2159-8290.CD-20-1285 PMC8487926

[CR36] Song Q, He X, Xiong Y, Wang J, Zhang L, Leung EL-H, Li G (2021) The functional landscape of golgi membrane protein 1 (GOLM1) phosphoproteome reveal GOLM1 regulating P53 that promotes malignancy. Cell Death Discov 7:42. 10.1038/s41420-021-00422-2 33649292 10.1038/s41420-021-00422-2 PMC7921442

[CR35] Sui T, Wang X, Li L, Liu J, Qiao N, Duan L, Shi M, Huang J, Yang H, Cheng G (2021) GOLM1 suppresses autophagy-mediated anti-tumor immunity in hepatocellular carcinoma. Signal Transduct Target Ther 6:335. 10.1038/s41392-021-00673-6 34531366 10.1038/s41392-021-00673-6 PMC8445956

[CR47] Sun Y, Gan Z, Wang X, Liu J, Zhong W, Zhang Z, Zuo J, Zhong H, Huang X, Yan Z, Cao Q (2024) Integrative metagenomic, transcriptomic, and proteomic analysis reveal the microbiota-host interplay in early-stage lung adenocarcinoma among non-smokers. J Transl Med 22:652. 10.1186/s12967-024-05485-0 38997719 10.1186/s12967-024-05485-0 PMC11245786

[CR1] Wu F, Fan J, He Y, Xiong A, Yu J, Li Y, Zhang Y, Zhao W, Zhou F, Li W, Zhang J, Zhang X, Qiao M, Gao G, Chen S, Chen X, Li X, Hou L, Wu C, Su C, Ren S, Odenthal M, Buettner R, Fang N, Zhou C (2021) Single-cell profiling of tumor heterogeneity and the microenvironment in advanced non-small cell lung cancer. Nat Commun 12:2540. 10.1038/s41467-021-22801-0 33953163 10.1038/s41467-021-22801-0 PMC8100173

[CR17] Xiang H, Pan Y, Sze MA, Wlodarska M, Li L, van de Mark KA, Qamar H, Moure CJ, Linn DE, Hai J, Huo Y, Clarke J, Tan TG, Ho S, Teng KW, Ramli MN, Nebozhyn M, Zhang C, Barlow J, Gustafson CE, Gornisiewicz S, Albertson TP, Korle SL, Bueno R, Moy LY, Vollmann EH, Chiang DY, Brandish PE, Loboda A (2024) Single-cell analysis identifies NOTCH3-mediated interactions between stromal cells that promote microenvironment remodeling and invasion in lung adenocarcinoma. Cancer Res 84:1410–1425. 10.1158/0008-5472.CAN-23-1183 38335304 10.1158/0008-5472.CAN-23-1183 PMC11063690

[CR48] Xu J, Zhang Y, Li M, Shao Z, Dong Y, Li Q, Bai H, Duan J, Zhong J, Wan R, Bai J, Yi X, Tang F, Wang J, Wang Z (2024) A single-cell characterised signature integrating heterogeneity and microenvironment of lung adenocarcinoma for prognostic stratification. EBioMedicine 102:105092. 10.1016/j.ebiom.2024.105092 38547579 10.1016/j.ebiom.2024.105092 PMC10990706

[CR37] Yamoah K, Johnson MH, Choeurng V, Faisal FA, Yousefi K, Haddad Z, Ross AE, Alshalafa M, Den R, Lal P, Feldman M, Dicker AP, Klein EA, Davicioni E, Rebbeck TR, Schaeffer EM (2015) Novel biomarker signature that may predict aggressive disease in African American men with prostate cancer. J Clin Oncol 33:2789–2796. 10.1200/JCO.2014.59.8912 26195723 10.1200/JCO.2014.59.8912 PMC4550692

[CR6] Zhang P, Zhang X, Cui Y, Gong Z, Wang W, Lin S (2023a) Revealing the role of regulatory T cells in the tumor microenvironment of lung adenocarcinoma: a novel prognostic and immunotherapeutic signature. Front Immunol 14:1244144. 10.3389/fimmu.2023.1244144 37671160 10.3389/fimmu.2023.1244144 PMC10476870

[CR20] Zhang N, Zhang H, Liu Z, Dai Z, Wu W, Zhou R, Li S, Wang Z, Liang X, Wen J, Zhang X, Zhang B, Ouyang S, Zhang J, Luo P, Li X, Cheng Q (2023b) An artificial intelligence network-guided signature for predicting outcome and immunotherapy response in lung adenocarcinoma patients based on 26 machine learning algorithms. Cell Prolif 56:e13409. 10.1111/cpr.13409 36822595 10.1111/cpr.13409 PMC10068958

[CR18] Zhang S, Liu Y, Sun Y, Liu Q, Gu Y, Huang Y, Zeng Z, Tang F, Ouyang Y (2024) Aberrant R-loop-mediated immune evasion, cellular communication, and metabolic reprogramming affect cancer progression: a single-cell analysis. Mol Cancer 23:11. 10.1186/s12943-023-01924-6 38200551 10.1186/s12943-023-01924-6 PMC10777569

[CR32] Zheng H, Long G, Zheng Y, Yang X, Cai W, He S, Qin X, Liao H (2022) Glycolysis-related SLC2A1 is a potential pan-cancer biomarker for prognosis and immunotherapy, Cancers (Basel) 14. 5344. 10.3390/cancers14215344 10.3390/cancers14215344 PMC9657346 36358765

