# Supplementary material for: Integrative analysis of genetic variability and functional traits in lung adenocarcinoma epithelial cells via single-cell RNA sequencing, GWAS, bayesian deconvolution, and machine learning
Source: Genes Genomics. 2025 Feb 24;47(4):435–68. doi: 10.1007/s13258-025-01621-2 (PMC12000210; doi:10.1007/s13258-025-01621-2)
Supplement: Supplementary file 1 — Supplementary Information 1. [file 13258_2025_1621_MOESM1_ESM.docx]

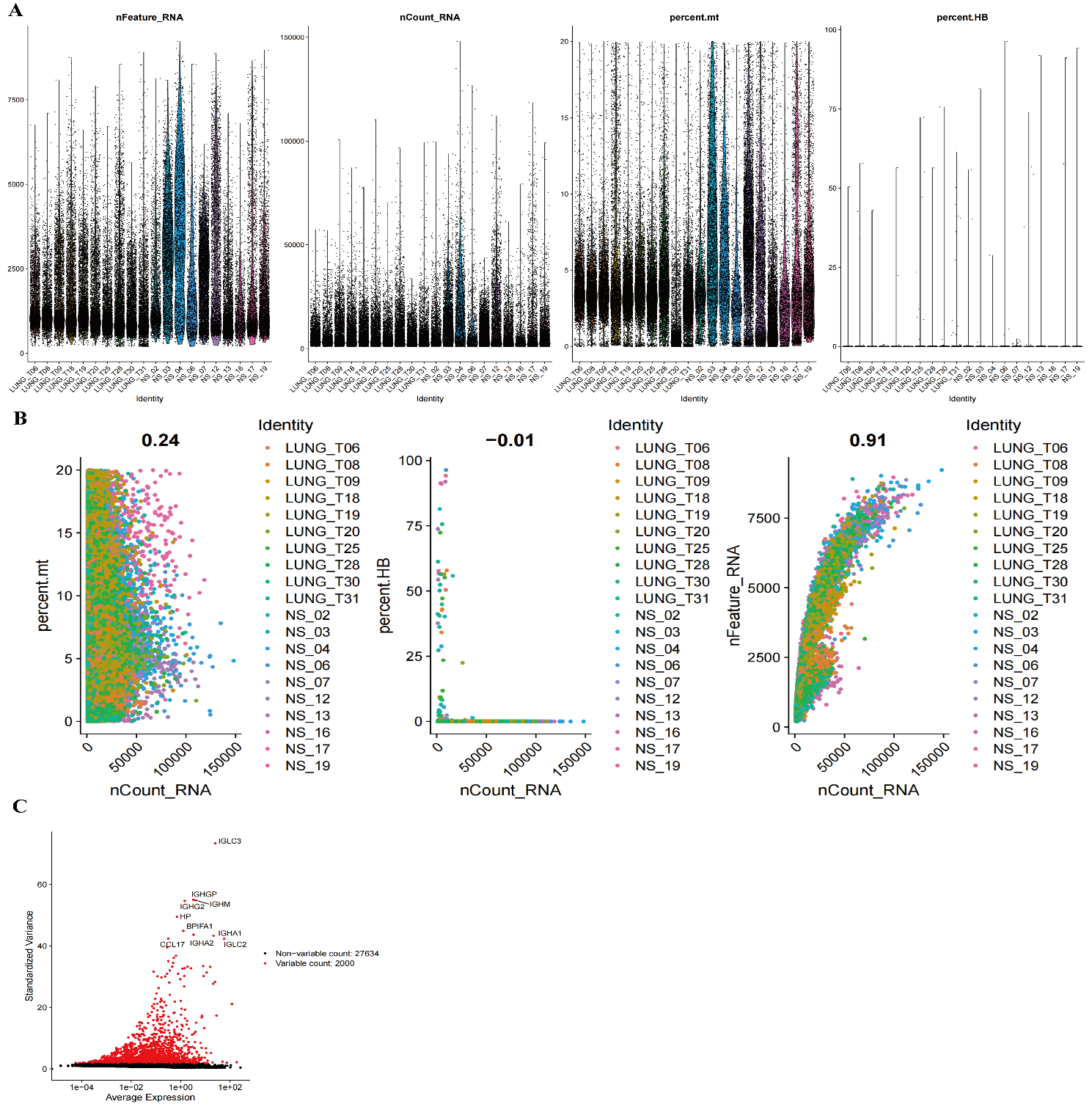


**Figure S1: Quality Control and Dimensionality Reduction Analysis (A)** This panel illustrates the distribution of RNA feature number (nFeature_RNA), RNA counts (nCount_RNA), mitochondrial gene proportion (percent.mt), and red blood cell gene proportion (percent.HB) across samples. Each data point represents a single cell, and different colors indicate different samples. The mitochondrial gene proportion (percent.mt) and red blood cell gene proportion (percent.HB) were used to assess data quality. **(B)** The correlation between RNA counts (nCount_RNA) and other metrics is displayed. The left plot shows a correlation of 0.24 between mitochondrial gene proportion and RNA counts, the middle plot shows a correlation of -0.01 between red blood cell gene proportion and RNA counts, and the right plot shows a correlation of 0.91 between RNA feature number and RNA counts. **(C)** This panel displays the results of the highly variable gene analysis, with red indicating genes with higher variability and black indicating genes with lower variability. The top 10 most variable genes are labeled, and these will be used for subsequent dimensionality reduction analysis.


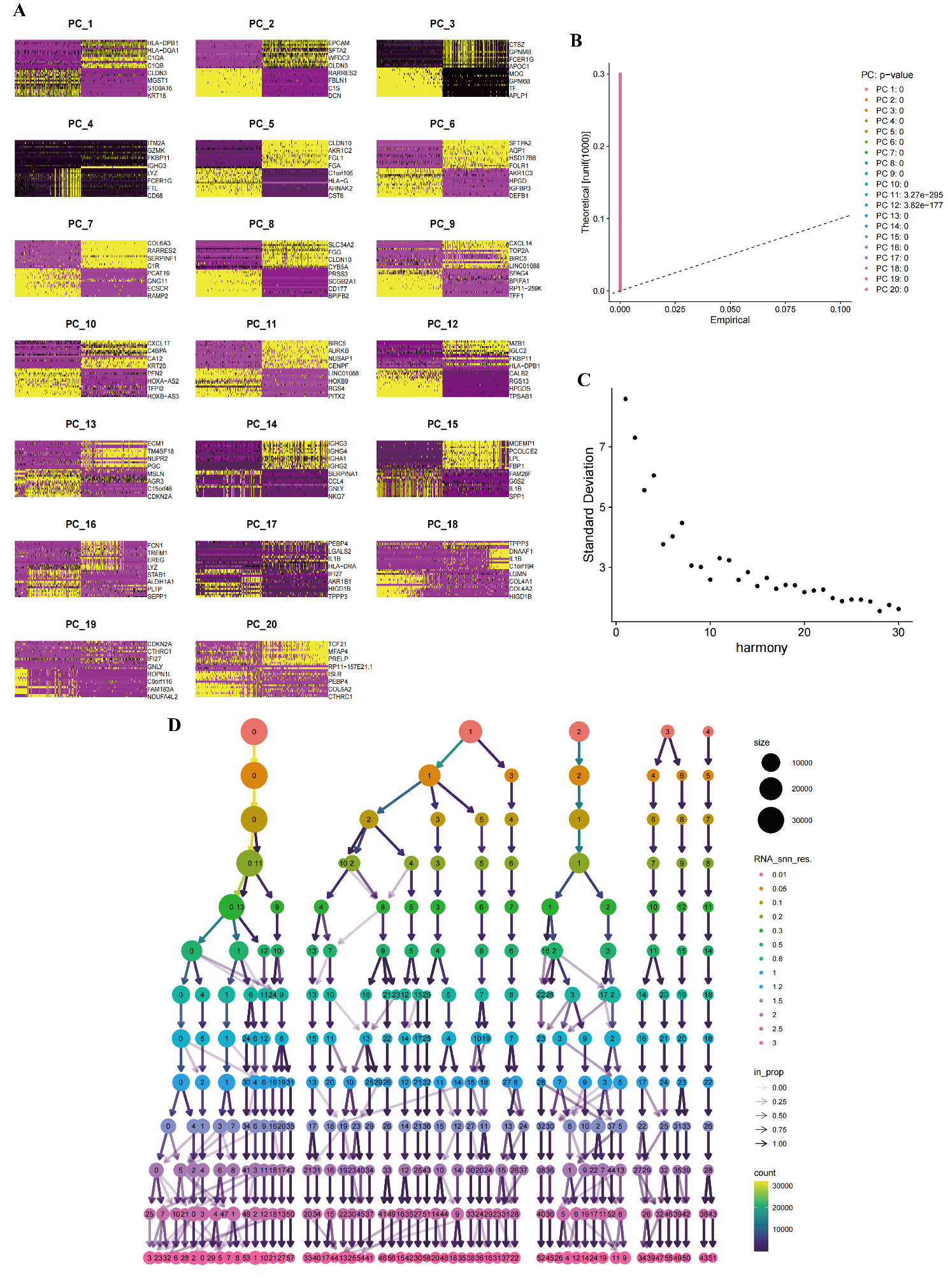


**Figure S2: PCA Feature Gene Analysis, Principal Component Significance Testing, and Clustering Results. (A)** Heatmap displaying the expression patterns of feature genes in the top 20 principal components (PC1–PC20). Each heatmap shows the gene expression profiles within different principal components, with yellow representing high expression and purple representing low expression. **(B)** The JackStraw test results showing the significance of the top 20 principal components. Principal components with smaller p-values are more significant in explaining data variation. The dashed line represents the theoretical p-value, and the red points indicate the most significant principal components. **(C)** The elbow plot displays the standard deviation of different principal components, helping to determine the optimal number of principal components for clustering analysis. Based on the plot, the top 7 principal components were selected for further analysis. **(D)** The Clustree plot illustrates the hierarchical relationships between cell clusters at different resolutions. Each circle represents a cell cluster at a given resolution, with color and size indicating the resolution and cluster size, respectively. The plot clearly demonstrates the hierarchical structure of cell clustering across multiple resolutions.


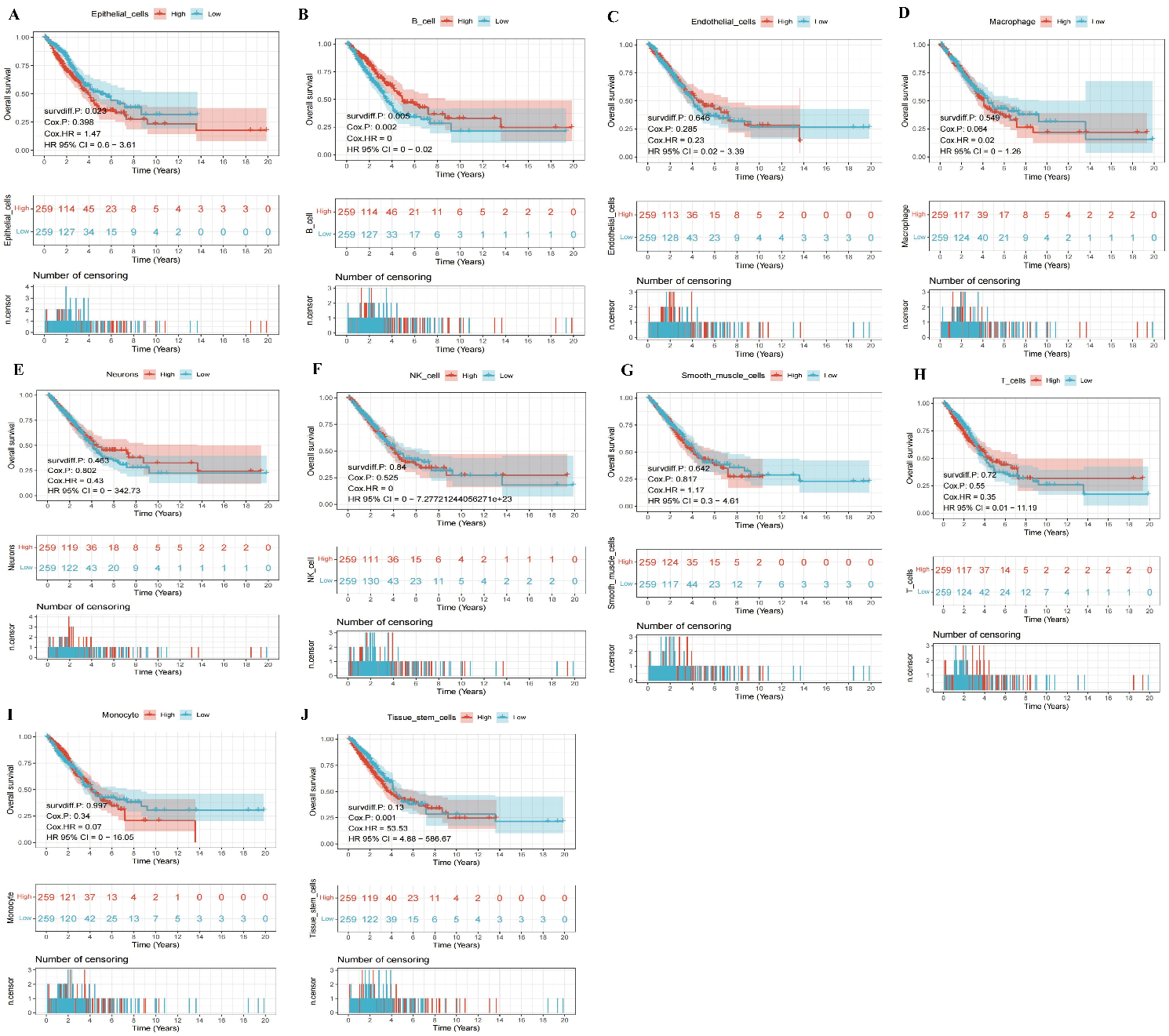


**Figure S3 Association Analysis Between Different Cell Types and Overall Survival** Each survival curve plot illustrates the relationship between high-risk (red) and low-risk (blue) groups for different cell types and their overall survival.


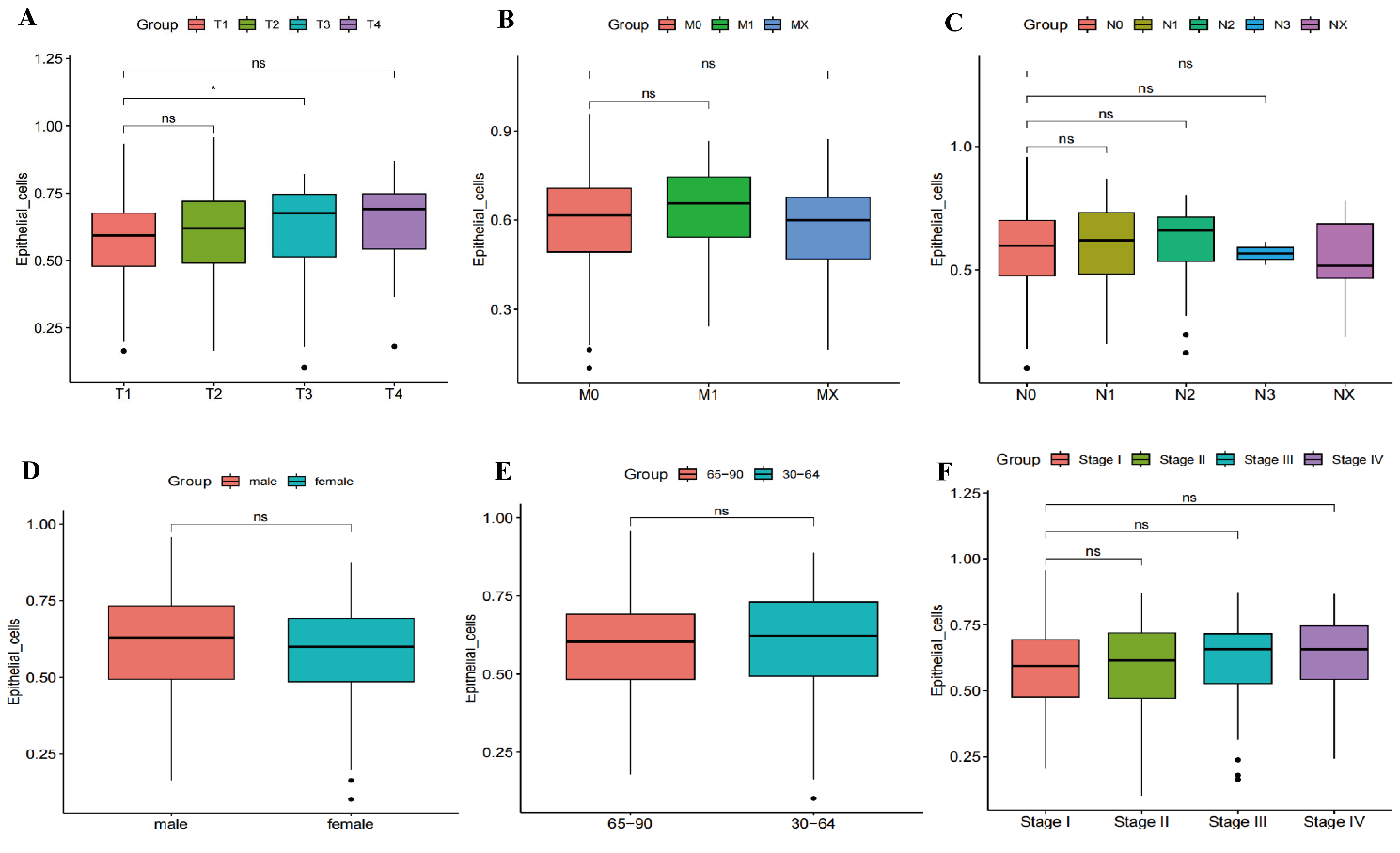


**Figure S4. Distribution of Epithelial Cells Across Different Clinical Characteristics** (A) Distribution of epithelial cells across different T stages (T1, T2, T3, T4). The analysis shows a significant difference between the T1 and T3 groups (p < 0.05), with no significant differences between other groups (ns). (B) Distribution of epithelial cells across different M stages (M0, M1, MX), with no significant differences (ns). (C) Distribution of epithelial cells across different N stages (N0, N1, N2, N3, NX), with no significant differences (ns). (D) Distribution of epithelial cells across gender groups (male, female), with no significant differences (ns). (E) Distribution of epithelial cells across different age groups (30–64 years, 65–90 years), with no significant differences (ns). (F) Distribution of epithelial cells across different clinical stages (Stage I, Stage II, Stage III, Stage IV), with no significant differences (ns).


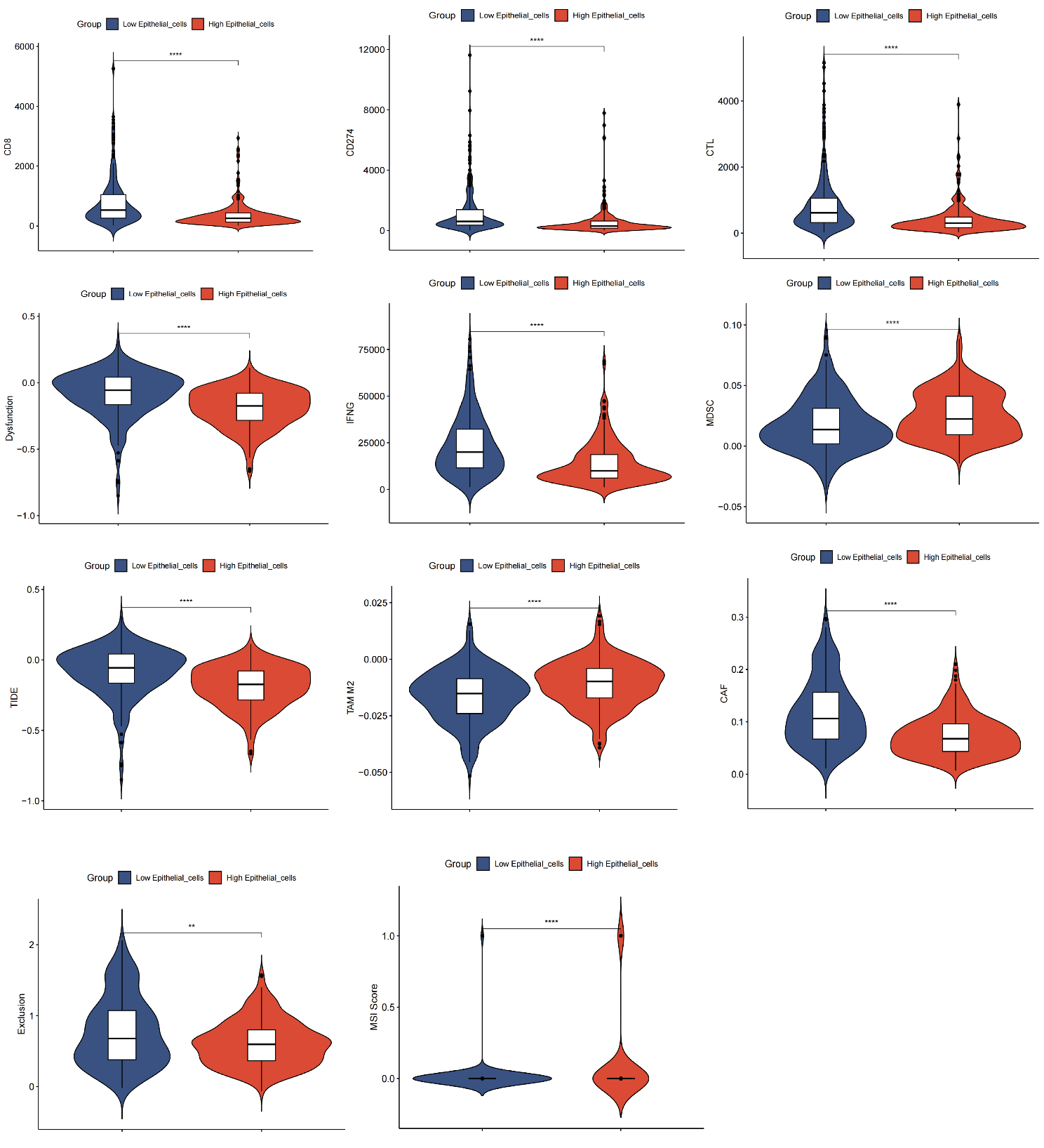


**Figure S5. Differential Analysis of Immune-Related Markers Between High and Low Epithelial Cell Expression Groups** This figure displays the significant differences in various immune-related markers between the high epithelial cell expression group (red) and the low epithelial cell expression group (blue).


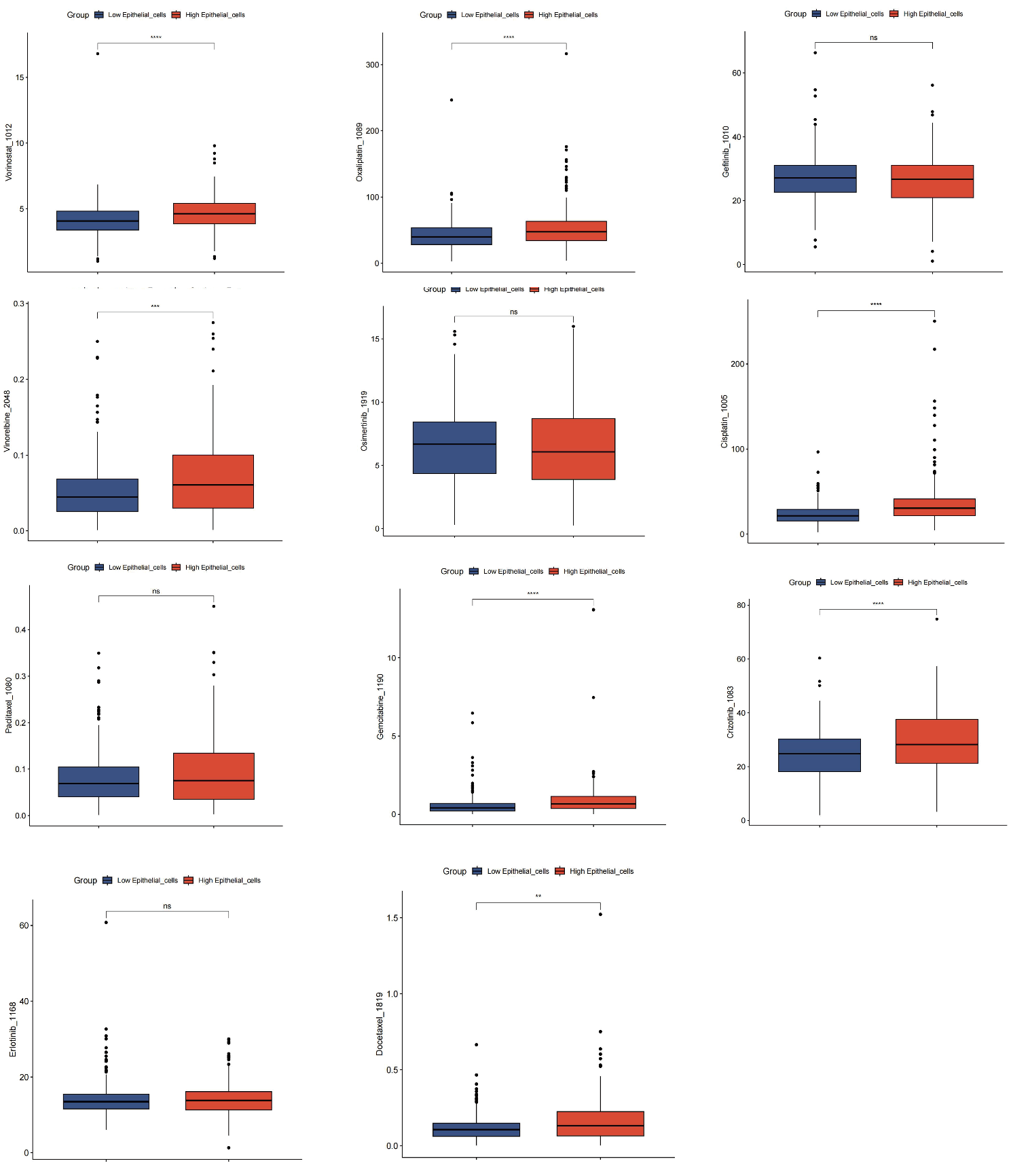


**Figure S6. Drug Response Prediction Analysis for Epithelial Cell Groups**This figure illustrates the differences in drug responses between the "Low Epithelial_cells" group and the "High Epithelial_cells" group across various drugs.
